# Animals have expanded the evolutionary legacy of unicellular ancestors in blood cells

**DOI:** 10.1073/pnas.2528110123

**Published:** 2026-05-28

**Authors:** Yosuke Nagahata, Yuji Nishimura, Ryota Kaitani, Jason Cheok Kuan Leong, Izumi Oda-Ishii, Hisanori Kohtsuka, Shinya Abe, Tasuku Ishida, Marina Carmona-Rivas, Sebastián R. Najle, Elena Casacuberta, Koichi Ikuta, Toru Miura, Michio Ogasawara, Naoki Irie, Yutaka Satou, Iñaki Ruiz-Trillo, Hiroshi Kawamoto

**Affiliations:** ^a^ Laboratory of Immunology, Institute for Life and Medical Sciences, Kyoto University, Kyoto 606-8507, Japan; ^b^ Department of Complexity of Life, Institut de Biologia Evolutiva (Consejo Superior de Investigaciones Científicas-Universitat Pompeu Fabra), Barcelona 08003, Spain; ^c^Japan Society for the Promotion of Science Overseas Research Fellow, Japan Society for the Promotion of Science, Tokyo 102-0083, Japan; ^d^ Research Center for Integrative Evolutionary Science, The Graduate University for Advanced Studies (SOKENDAI), Hayama, Kanagawa 240-0193, Japan; ^e^ Department of Zoology, Graduate School of Science, Kyoto University, Kyoto 606-8502, Japan; ^f^ Misaki Marine Biological Station, School of Science, The University of Tokyo, Miura, Kanagawa 238-0225, Japan; ^g^ Laboratory of Immune Regulation, Institute for Life and Medical Sciences, Kyoto University, Kyoto 606-8507, Japan; ^h^ Department of Homeostatic Medicine, Medical Research Institute, Institute of Science Tokyo, Tokyo 113-8510, Japan; ^i^ Department of Biomedical Sciences, Faculty of Medicine and Health Sciences, Universitat Internacional de Catalunya, Barcelona 08195, Spain; ^j^ Department of Biology, Graduate School of Science, Chiba University, Chiba 263-8522, Japan; ^k^ Institució Catalana de Recerca i Estudis Avançats, Barcelona 08010, Spain

**Keywords:** blood cell, evolution, unicellular organism, mast cell, thymus

## Abstract

Blood cells are common and unique to animals, enabling them to address critical challenges of defense and transport. Thus, their evolution represents a defining innovation in metazoan multicellular life. However, their evolutionary trajectory about how blood cells emerged and diversified throughout animal history remains unclear. Here, we present a combination of bioinformatics and functional data that demonstrate that the metazoan blood cell program most likely originated through the repurposing of an ancestral premetazoan toolkit governed by *Fos*. This primordial program established the macrophage-like initial blood cells at the metazoan root. Then, the first lineage bifurcation at the origin of Bilateria drove the emergence of a specialized mast/killer lineage, characterized by acquisition of granular proteases for antiparasitic defense. Subsequent deuterostome/vertebrate innovations branched T/NK and erythrocyte/thrombocyte lineages from mast cells while B cells derived from macrophages. Our data also show that a prototypic thymus formed at the gill edges of a chordate ancestor. In line with the evolutionary history, the modern hematopoietic pathway shows a vestige of the phylogeny; differentiation potentials of phylogenetically old cell lineages expressing *Fos* such as macrophages and mast cells are widely retained, and ancient HSCs with limited lineage potentials have been inherited as origo-lineage progenitors. Our framework provides the history of blood cells showing an adaptive innovation built upon ancient unicellular foundations.

## Significance

This study provides a transcriptome-based model for the evolutionary history of animal blood cells, from emergence to divergence. The initial blood cells emerged in early animals inheriting unicellular ancestors’ features, followed by the evolution of mast cells in bilaterian ancestors to fight against parasites. Thereafter, in deuterostome/vertebrate ancestors, T/NK cells and erythrocytes/thrombocytes arose from the ancestral mast cells, while B cells evolved from the ancestral macrophages. Along with diversification of blood cell lineages, prototypic thymus formed at the gill edges in chordate ancestors. We finally found a vestige of the evolutionary history in murine hematopoiesis by detecting widely retained macrophage and mast cell potential. These findings shed light on a reciprocal relationship between the evolutionary history and current development pathways.

The transition from unicellular organisms to animal multicellularity created fundamental challenges for animal life, chief among them the need for internal defense and resource transport. The evolution of specialized, circulating cell populations—the basis of blood and immunity—was a critical innovation to solve these problems. Hence, blood cells are unique to animals, and they are widely shared among animals, even in cnidarian species (1). Another innovation is diversification of blood cells. In vertebrates, a diverse array of blood cell lineages orchestrates a multifaceted defense against pathogens: macrophages, B cells, T cells, natural killer (NK) cells, innate lymphoid cells (ILCs), dendritic cells (DCs), and granulocytes such as mast cells (2–7). Beyond immune function, erythrocytes, the oxygen transporters of the body, represent another indispensable blood cell lineage. However, the evolutionary logic governing the diversification of these cell types remains unresolved.

We recently found that a phagocytic program was inherited from unicellular organisms to vertebrates, and the earliest blood cells were likely macrophage-like cells (8), but that study did not reveal how blood cells diversified from the ancestral macrophages lacking a phylogenetic tree of blood cell lineages or protostome species. Although T cells, B cells, and erythrocytes are considered unique in vertebrates (9–11), and a recent study in deuterostome species supports the existence of prototypic cytotoxic activity in the ancestor of Deuterostomia (12–14), a comprehensive phylogeny of blood cell lineages remains unveiled. Furthermore, the broader evolutionary relationship between blood cells and non-blood cells, and the precise emergence of the initial blood cells in metazoan ancestors, have not been predicted in detail.

Here, by improving our method to compare transcriptome data across diverse species, from vertebrates to unicellular organisms (*SI Appendix*, Fig. S1 *A*–*D*), we constructed a comprehensive phylogenetic tree of animal cell lineages, and approached the evolutionary history about diversification of these blood cell lineages.

## Results

### Macrophage-Like Blood Cells Evolved at the Onset of Metazoa, Inheriting Premetazoan Unicellular Features.

**Fig. 1.**
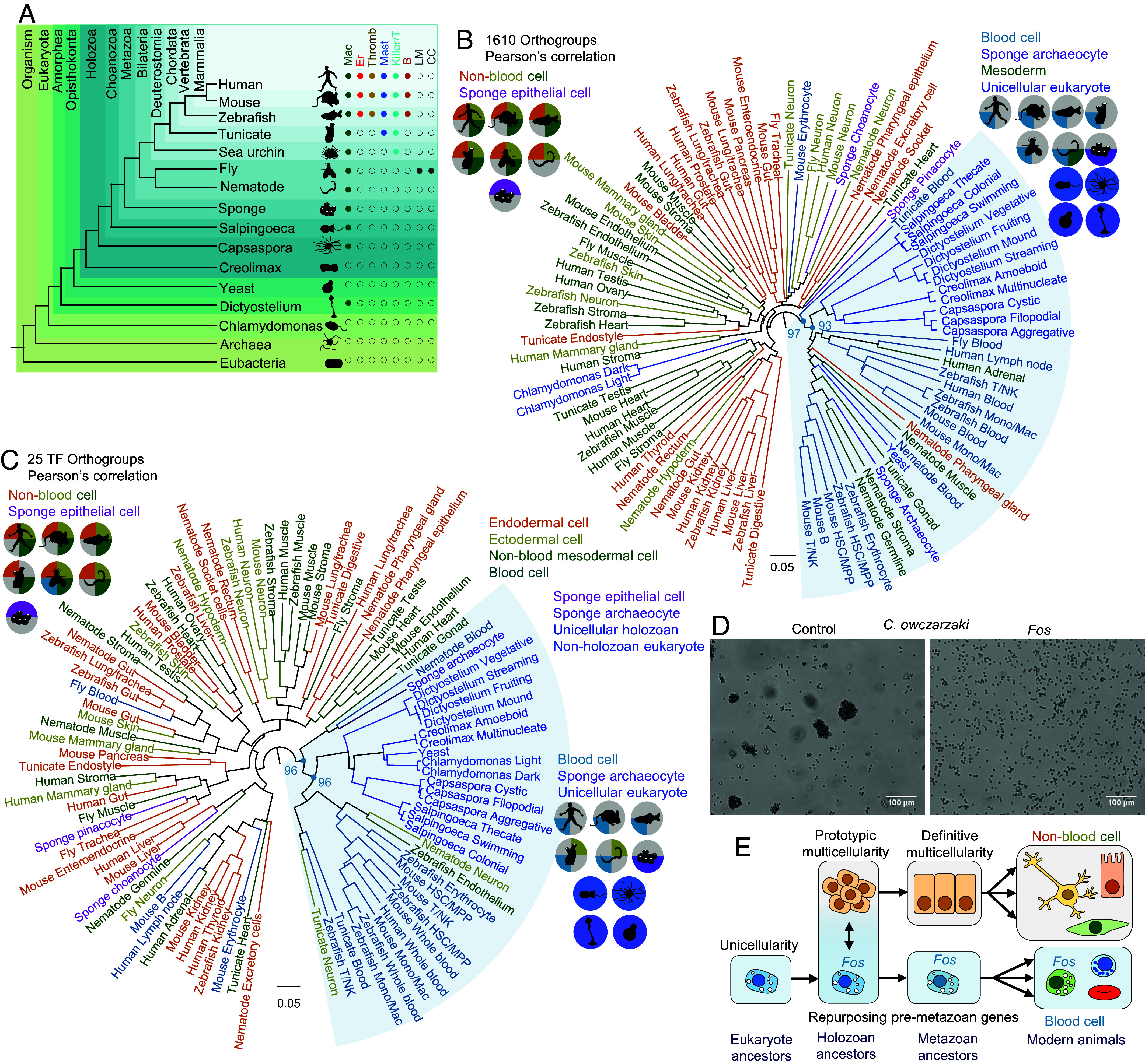
T Macrophage-like blood cells evolved at the onset of Metazoa, inheriting premetazoan unicellular features. (*A*) Organisms analyzed in this study and their blood cell lineages. Vertebrates have multiple hemopoietic lineages, but fewer lineages are found in invertebrate animals. Macrophages are common in almost all animals, but killer cells with cytotoxicity are restricted to more narrow clades. Some unicellular eukaryotes are known to behave like macrophages with phagocytic potentials (8, 16, 17). (*B* and *C*) Neighbor-joining phylogenetic trees of animal cell lineages and unicellular organisms based on 1,610 Orthogroups (*B*) or highly variable 25 TFs (*C*). Nodal support numbers of adjusted unbiassed bootstrap values at key bifurcations are shown with colored numbers and circles. Cell lineages frequently included in each clade are shown with colored quarter circles. The blood cell clade is highlighted with blue background. (*D*) Representative images of *C. owczarzaki* with enforced expression of *Fos* (n = 3). (*E*) Schematic illustration of evolution of blood cells from unicellular ancestors. Er, erythrocyte; Mac, macrophage; Mono, monocyte; Neut, neutrophil; Thromb, thrombocyte.

First, we addressed the emergence of the initial blood cells in animals by conducting a comprehensive analysis of transcriptome data from diverse whole-body cell lineages. Our dataset included human (*Homo sapiens*), mouse (*Mus musculus*), zebrafish (*Danio rerio*), an early-branching chordate (tunicate, *Ciona robusta*/*Ciona intestinalis*, type A), fly (*Drosophila. melanogaster*), a nematode (*Caenorhabditis elegans*), and a sponge (*Amphimedon queenslandica*) (Fig. 1*A* and *SI Appendix*, Fig. S1*A*). We also included unicellular eukaryotes, such as the close relatives of animals, *Salpingoeca rosetta*, *Capsaspora owczarzaki*, and *Creolimax fragrantissima*, alongside *Saccharomyces cerevisiae*, *Dictyostelium discoideum*, and *Chlamydomonas reinhardtii*, served as outgroups (Fig. 1*A*). Our phylogenetic tree showed a clade containing blood cells, sponge archaeocytes which circulate throughout the body and have phagocytic activity like blood cells (15), and unicellular organisms (Fig. 1*B* and *SI Appendix*, Fig. S2*A*). Our inferred tree suggests that the initial blood cells emerged in an early animal ancestor inheriting unicellular ancestors’ features. Moreover, correlation values, especially those of *S. rosetta* and *C. owczarzaki* suggests that the unicellular ancestors were similar to macrophages among vertebrate blood cell lineages (*SI Appendix*, Fig, S2*A*). To further evaluate whether blood cells and unicellular organisms are transcriptionally similar, we examined the evolutionary oldness of genes expressed in each cell lineage using gene age scores. Compared to nonblood cell lineages, blood cells more profoundly expressed ancient genes originated from unicellular ancestors and expressed fewer relatively novel genes of metazoan or bilaterian origin. This provides further support that blood cells were transcriptionally similar to unicellular organisms (*SI Appendix*, Fig. S1 *E* and *F*).

Next, to rule out convergent evolution—where similar lifestyles might independently drive similar gene expression—we focused on transcription factors (TFs) whose expression profiles also identify cell lineages (18). Because TFs govern regulatory networks rather than executing terminal functions like enzymes, they serve as more reliable and complementary markers of homology. The TF-based phylogeny reinforced our whole-transcriptome findings: Animal blood cells and unicellular holozoans share a conserved regulatory core (Fig. 1*C* and *SI Appendix*, Fig. S2*B*). The two phylogenetic trees collectively suggest that blood cells evolved in early animals inheriting unicellular features (Fig. 1*E*). To pinpoint the key TFs that underpinned the emergence of the initial blood cells, we identified those commonly expressed or repressed across the blood cells of human, mouse, zebrafish, tunicate, fly, nematode, sponge, and filopodial/amoeboid stage of unicellular *C. owczarzaki* (*SI Appendix*, Fig. S3*A*). Of note, *Fos* and *Stat1-5* homologs were highly expressed among all animal blood cells analyzed, suggesting that their pivotal role in determining the blood cell lineage in metazoan ancestors. *Cebpa* was another TF which highly expressed among all bilaterian blood cells. Its homolog was not highly expressed in sponge archaeocytes, but filopodial/amoeboid stage of *C. owczarzaki* highly expressed its homolog. Among these TFs, *Fos* is known to have a potential to convert fibroblasts to blood cells (19), which supports its pivotal role to determine the blood cell lineage. *Fos* also exhibited high expression in the swimming or filopodial/amoeboid stages of unicellular holozoan species *S. rosetta* and *C. owczarzaki*. To test whether *Fos* is the functional driver of this “blood-like” state, we performed enforced expression experiments in the close animal relative *C. owczarzaki*. Overexpression of *Fos* actively inhibited cellular aggregation and maintained cells in an amoeboid state (Fig. 1*D* and *SI Appendix*, Fig. S3 *B*–*E*). These results suggest that the blood cell genetic program characterized by *Fos* expression can be traced back to the common ancestor of animals and holozoan unicellular organisms (Fig. 1*E*).

### Divergence of Mast/Killer Lineage Cells Occurred at the Origin of Bilateria.

**Fig. 2.**
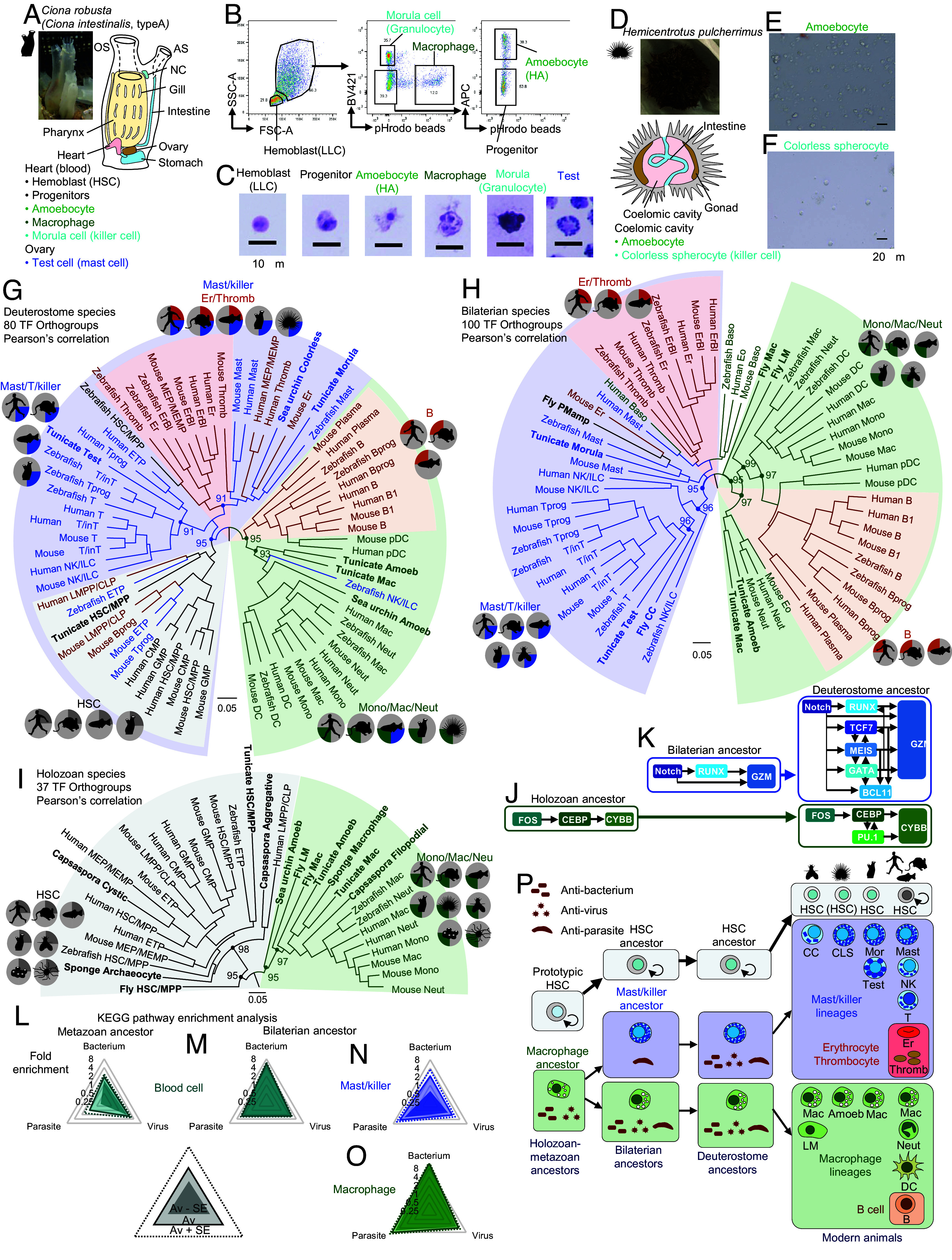
Divergence of mast/killer lineage cells occurred at the origin of Bilateria. (*A*) An image and a diagram of tunicate. Analyzed blood cell lineages are listed. (*B*) Collected tunicate blood cells were analyzed by flow cytometry. We regarded autofluorescence-negative large cells as progenitors based on their transcriptional similarity to hemoblasts. Hemoblasts, amoebocytes, and morula cells are also called lymphocyte-like cells (LLC), hyaline amoebocytes (HA), and granulocytes, respectively. (*C*) Representative images of tunicate blood cells and test cells with Wright Giemsa stain. (*D*) An images and a diagram of sea urchin. (*E* and *F*) Representative images of sea urchin amoebocytes (*E*) and CLSs (*F*). (*G*–*I*) Neighbor-joining phylogenetic trees of blood cell lineages based on TFs among deuterostome (*G*), bilaterian (*H*), and holozoan (*I*) species. Nodal support numbers of adjusted unbiassed bootstrap values at key bifurcations are shown with colored numbers and circles. Cell lineages included in each clade are shown with colored quarter circles. Tunicate, sea urchin, fly, and sponge blood cells and *C. owczarzaki* are shown in bold characters. The macrophage, mast/killer cell, B cell, erythrocyte/thrombocyte, and HSC/progenitor clades are highlighted with green, blue, orange, red, and gray background, respectively. In the deuterostome species tree (*G*), eosinophils/basophils were excluded. In the bilaterian species tree (*H*), HSCs/progenitors were excluded. In the holozoan species tree (*I*), mast/killer cell lineages and B cell lineages were excluded, and HSC/progenitor and macrophage lineages were analyzed. (*J* and *K*) Estimated TF networks in ancestral macrophage lineages (*J*) and mast/killer lineages (*K*) based on expression data and TF binding sites in multiple organisms. (*L*–*O*) KEGG pathway analysis for genes estimated to have been expressed in ancestral blood cells (*L* and *M*), mast/killer cells (*N*), and macrophages (*O*). Those in metazoan (*L*) and bilaterian ancestors (*M*–*O*) were estimated. Fold enrichment scores for infectious disease were analyzed, and mean values ± SE of bacterial, viral, and parasitic infection are represented. (*P*) Schematic illustration of evolution of blood cell lineages from a holozoan ancestor. Amoeb, amoebocyte; Baso, basophil; Bprog, B-cell progenitor; CLP, common lymphoid progenitor; Eo, eosinophil; Er, erythrocyte; GMP, granulocyte-monocyte progenitor; Mac, macrophage; Mono, monocyte; MPP, multipotent progenitor; Neut, neutrophil; PMamp, plasmatocyte expressing antimicrobial peptides; Thromb, thrombocyte; Tprog, T-cell progenitor.

By constructing the phylogenetic trees covering both animals and unicellular organisms and both blood cells and non-blood cells, we succeeded in estimating the initial blood cells of animals. Next, to delineate the evolutionary divergence of the blood cell lineages, we analyzed transcriptome data from diverse animal blood cell lineages, and constructed phylogenetic trees based on TFs in stepwise and reverse chronological order: deuterostome species, bilaterian species, and holozoan species. In addition to public data of human, mouse, zebrafish, fly, sponge, and *C. owczarzaki*, we obtained novel transcriptome data of tunicate (*C. robusta*) and sea urchin (*Hemicentrotus pulcherrimus*) to have a broader taxon-sampling that covers major evolutionary nodes (Fig. 2 *A*–*F* and *SI Appendix*, Fig. S4 *A*–*H*). Tunicates are known to have macrophages and cytotoxic killer cells (morula cells) in their colorless blood, as well as mast cell–like cells (test cells) in the ovary (Figs. 1*A* and 2 *A*–*C*) (12, 13, 20). Sea urchins have macrophage-like amoebocytes and cytotoxic colorless spherocytes (CLSs) (Figs. 1*A* and 2 *D*–*F*) (21, 22). First, the tree of deuterostome species showed that macrophages/amoebocytes of vertebrate, tunicate, and sea urchin clustered together in the same clade (Fig. 2*G* and *SI Appendix*, Fig. S5 *A*–*C*), validating the methodology in this analysis. Interestingly, tunicate and sea urchin killer cells grouped in a clade of vertebrate mast cells, while tunicate mast cells (test cells) grouped with vertebrate cytotoxic T/NK cells (Fig. 2*G* and *SI Appendix*, Fig. S5*B*). One probable explanation is that cytotoxic killer cells and mast cells came from the same ancestral deuterostome blood cell. Moreover, while the similarity between T/killer cells and mast cells has been underexplored, we identified that granzymes and mast cell proteases (MCPTs), which are functional proteins specific to T/NK cells and mast cells in vertebrates, shared similar amino acid sequences (*SI Appendix*, Fig. S4*I*). Importantly, homologs of these proteins were highly expressed in tunicate and sea urchin killer/mast lineage cells (*SI Appendix*, Fig. S4 *J* and *K*). These data demonstrated a transcriptional similarity between killer cells and mast cells in vertebrates, tunicate, and sea urchin. The phylogenetic trees also elucidated the evolutionary history of vertebrate-unique lineages: erythrocytes, thrombocytes, and B cells. The trees showed that the erythrocyte/thrombocyte lineage was close to the mast/killer cell lineage rather than the macrophage lineage suggesting that they emerged within the mast/killer clade (Fig. 2*G* and *SI Appendix*, Fig. S5*B*). On the other hand, the B lineage seems to have emerged within the macrophage clade (Fig. 2*G* and *SI Appendix*, Fig. S5*B*). Shared antigen-presenting function among macrophages, DCs, and B cells supports the emergence of B cells within the macrophage clade.

Having established two major lineages (macrophages and mast/killer cells) within deuterostome blood cells, we extended our investigation to a bilaterian ancestor by incorporating gene expression profiles from protostome *D. melanogaster* (fly) blood cell lineages (23). The phylogenetic analysis of bilaterian blood cell lineages showed that fly macrophages and lamellocytes (LMs) were transcriptionally similar to deuterostome macrophages, while fly crystal cells (CCs) were similar to deuterostome mast/killer cells (Fig. 2*H* and *SI Appendix*, Fig. S5*D*). The similarity between fly LMs and macrophages was further confirmed by their shared high expression of phagocytosis-related genes (*SI Appendix*, Fig. S4*L*). The similarity between fly CCs and mast/killer cells was also supported by their high expression of a granzyme homolog (*SI Appendix*, Fig. S4*M*).

Finally, we constructed the tree of holozoan species focusing on hematopoietic stem cells (HSCs)/progenitors and macrophage lineages (Fig. 2*I* and *SI Appendix*, Figs. S4*N* and S5*E*). Our results showed that filopodial/amoeboid stage of *C. owczarzaki* was similar to macrophage lineages of animals, while its aggregative and cystic stages were similar to HSCs/progenitors of animals. This suggests that prototypic HSCs or immature status of macrophages can be traced back to a holozoan ancestor. In other words, a prototypic blood cell differentiation was inherited from a premetazoan unicellular ancestor. Collectively, the phylogenetic trees of blood cell lineages suggest that differentiation from immature cells to macrophage-like phagocytes was a core blood cell feature inherited from a unicellular ancestor, and that the first lineage diversification occurred in a bilaterian ancestor creating ancestral mast cells which further diversified into T cells, NK cells, erythrocytes, and thrombocytes (Fig. 2*P*).

### Estimated TF Networks and Function of Ancestral Blood Cells.

Next, we analyzed TF expression profiles in detail to reveal lineage-specific signatures. *Cebpa/b/d/e* and *Spi1* homologs were highly expressed in the deuterostome macrophage lineages, while *Bcl11a/b*, *Gata1-6*, *Hes1/2*, *Meis/Pknox*, and *Tcf7/Lef1* homologs were highly expressed in the mast/killer cell lineages (*SI Appendix*, Fig. S6 *A*–*D* and *G*–*I*). Among these TFs, *Cebpa,* and *Spi1* are known to be important in macrophage/neutrophil differentiation (24, 25), while *Bcl11b*, *Gata3*, and *Tcf7* are important in T cell differentiation (26–29), supporting the accuracy of our analysis. Mouse has four T*cf7/Lef1* homologs, and *Tcf7* and *Tcf7l1* were expressed in T/NK cells and mast cells, respectively (*SI Appendix*, Fig. S6 *G* and *J*). There are also five *Meis/Pknox* homologs in mice, and *Meis1*, *Meis2*, and *Meis3* were expressed in HSC/MPP/Thrombocytes, mast cells, and T/NK cells, respectively (*SI Appendix*, Fig. S6 *G* and *J*). This suggested that gene duplications and selective expression of each duplicated TF enabled vertebrates to acquire various lineages. Comparisons extending to fly cells revealed that *Hes1/2* expression was conserved across deuterostome mast/killer cell lineages and fly CCs (*SI Appendix*, Fig. S6 *A*, *E*, and *F*). This, combined with the established role of Notch signaling in T cell, mast cell, and fly CC differentiation (30–36), suggested that the Notch signaling played a crucial role in the differentiation of mast/killer cells from bilaterian ancestors. We validated that these TF expressions were not just coincidences by identifying TF binding sites at promoter regions of TFs and functional genes, CYBB (NOX2) and granzymes (*SI Appendix*, Fig. S7 *A*–*D*). Together with TF expression profiles of various animal blood cells, these analyses supported the homology of cell lineages suggested by phylogenetic tress and also enabled us to estimate probable TF networks in ancestral blood cells (Fig. 2 *J* and *K* and *SI Appendix*, Fig. S7 *E* and *F*). As for TFs determining HSC programs inherited from a holozoan ancestor, we identified some candidates which were highly expressed in both various animal HSCs and HSC-like aggregative/cystic *C. owczarzaki* (SI *Appendix*, Fig. S6*K*). Among them, we checked the function of *Myb* in *C. owczarzaki*, and verified that it promoted cell proliferation (*SI Appendix*, Fig. S6*L*). Thus, it was supported that prototypical HSCs were formed based on a premetazoan proliferative state (Fig. 2*P*).

To further investigate the functional evolution of ancestral blood cells, we performed KEGG pathway enrichment analysis by selecting genes highly expressed in human blood cells and whose homologs were also highly expressed in blood cells of other animals. Among biological pathways, we focused on infectious diseases. Our analysis suggested that the initial metazoan blood cells were primarily equipped to combat bacteria and viruses, but not parasites (Fig. 2*L* and *SI Appendix*, Fig. S7*G*). In contrast, the blood cells of bilaterian and descendant ancestors demonstrated the capacity to fight against all three classes of pathogens (Fig. 2*M* and *SI Appendix*, Fig. S7 *G* and *H*). We then estimated the function of ancestral macrophage and mast/killer lineage cells separately (Fig. 2 *N* and *O*). It was shown that the bilaterian ancestor’s mast/killer cells mainly fought against parasites (Fig. 2*N* and *SI Appendix*, Fig. S7*G*), which was compatible with antiparasitic function of fly CCs and mast cells (7, 23, 37). This finding suggested that the emergence of mast/killer lineage cells in a bilaterian ancestor conferred a significant advantage in the fight against parasites (Fig. 2*P*). Moving forward in evolutionary time, our analysis suggested that the mast/killer lineage cells of deuterostome and vertebrate ancestors further acquired antibacterial and antiviral functions (*SI Appendix*, Fig. S7 *G*, *I*, and *J*). The acquisition of defensive capabilities, particularly the specialized antiparasitic function associated with the mast/killer lineage, implies the adaptive pressures that shaped blood cell evolution.

### A Prototypic Thymus Formed at Gill Edges in a Chordate Ancestor.

Next, we addressed the evolution of a hematopoietic environment especially for the T/NK/mast lineage, and attempted to find whether tunicates possess a thymus-like organ. We hypothesized that tunicate gills might share some features with vertebrate thymus. This hypothesis is predicated on several observations: i) lamprey gills display similarities to jawed vertebrate thymic epithelium, such as the expression of Notch ligands and *Foxn1* homologs (38–40), ii) the thymus of teleost and cartilaginous fishes are located near the gills (40), and iii) the murine thymus where T cells differentiate develops from the pharyngeal pouch (41, 42).

**Fig. 3.**
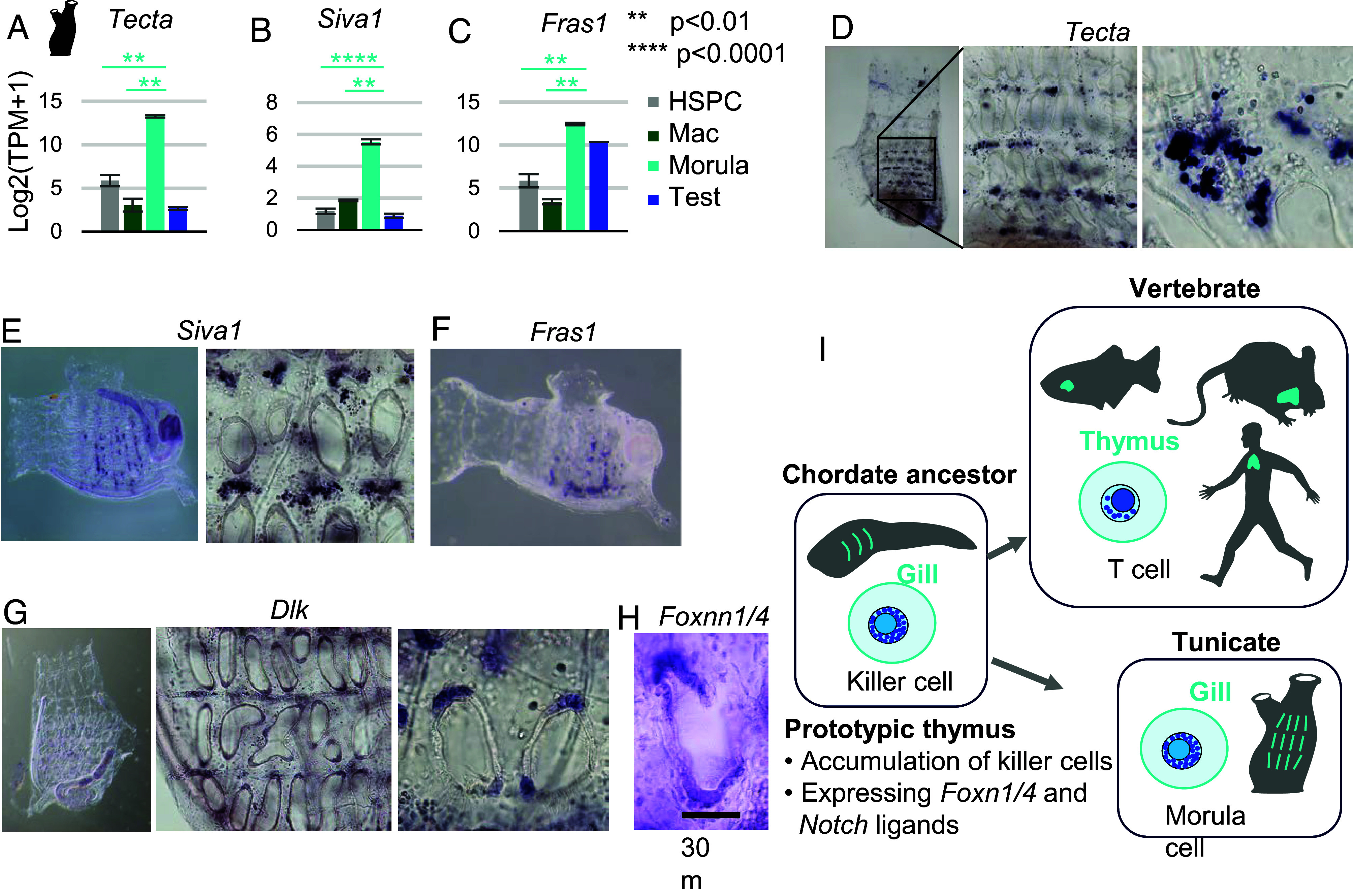
A prototypic thymus formed at gill edges in a chordate ancestor. (*A*–*F*) Expression levels with SE (*A*–*C*) and WISH images (*D*–*F*) of *Tecta*/ENSCING00000010439 (*A* and *D*), *Siva1*/ENSCING00000014939 (*B* and *E*), and *Fras1*/ENSCING00000000284 (*C* and *F*) which are highly (>10-fold) expressed in tunicate morula cells. The boxed area of the *Left* image in *D* is enlarged in the *Middle* image. The *Right* images in *D* and *E* show the gill edge of other individuals. Original WISH images of *Fras1* and other images of *Siva1* are available in the ghost database (http://ghost.zool.kyoto-u.ac.jp/cgi-bin/gb2/gbrowse/kh/). (*G* and *H*) Images of WISH experiments. Data of *Dlk* (*G*) and *Foxn1/4* (*H*) genes are shown. (*I*) Schematic illustration of thymus evolution in Chordata.

To test this hypothesis, we performed whole-mount in situ hybridization (WISH) experiments. We first identified 72 highly expressed marker genes (>10-fold) in tunicate morula cells. Among the 72 genes, we obtained WISH data of two genes (*Tecta*/ENSCING00000010439, and *Siva1*/ENSCING00000014939), and they showed that morula cells accumulated in the gill (Fig. 3 *A*, *B*, *D*, and *E*). Two other marker genes were available in the public tunicate database (http://ghost.zool.kyoto-u.ac.jp/default_ht.html) (43, 44), and one of the two (*Fras1*/ENSCING00000000284/KY21.Chr13.293) also showed that morula cells accumulated in the gill (Fig. 3 *C* and *F*). We also observed extravascular infiltration of some blood cells into the gill, with morula cells forming discernible clusters near the edges of the gill slits (Fig. 3 *D* and *E*, *Right*). Consistent with our hypothesis, the edges of the gill slits expressed the Notch ligand *Dlk* as previously reported (Fig. 3*G*) (45), and tunicate morula cells and test cells highly expressed *Hes1/2* homologs, which are downstream genes of Notch signaling (*SI Appendix*, Fig. S6*A*). Crucially, we identified expression of a *Foxn1/4* homolog, a pivotal TF in thymus development (46, 47), within these same gill slit edges (Fig. 3*H*). Collectively, our results indicate that the edge of the gill in tunicates is likely a homologous organ to the thymus, and suggest that a prototypic thymus emerged at the gill edges in chordate ancestors. Given that gills constitute a primary interface between the internal and external environments, serving as a common entry point for pathogens, the development or accumulation of cytotoxic cells within the gill would have conferred a significant defensive advantage in ancestral chordates.

### Retention of Macrophage and Mast Cell Potential in Hematopoietic Differentiation Pathways as a Vestige of the Evolutionary History.

Finally, we examined whether a vestige of the common evolutionary pathway of T/killer cells and mast cells could be detected in mouse hematopoiesis. In the conventional model of blood cell differentiation, mast cell potential is thought to be absent in lymphoid lineage progenitors, such as lympho-myeloid primed progenitors (LMPPs) and T-cell progenitors (48). However, a previous report suggested that T-cell progenitors retain mast cell potential (49). Given the close evolutionary link identified between T cells and mast cells in our phylogenetic analyses, we hypothesized that mast-cell potential could be detected in the lymphoid branch, especially in the T-cell differentiation pathway.

**Fig. 4.**
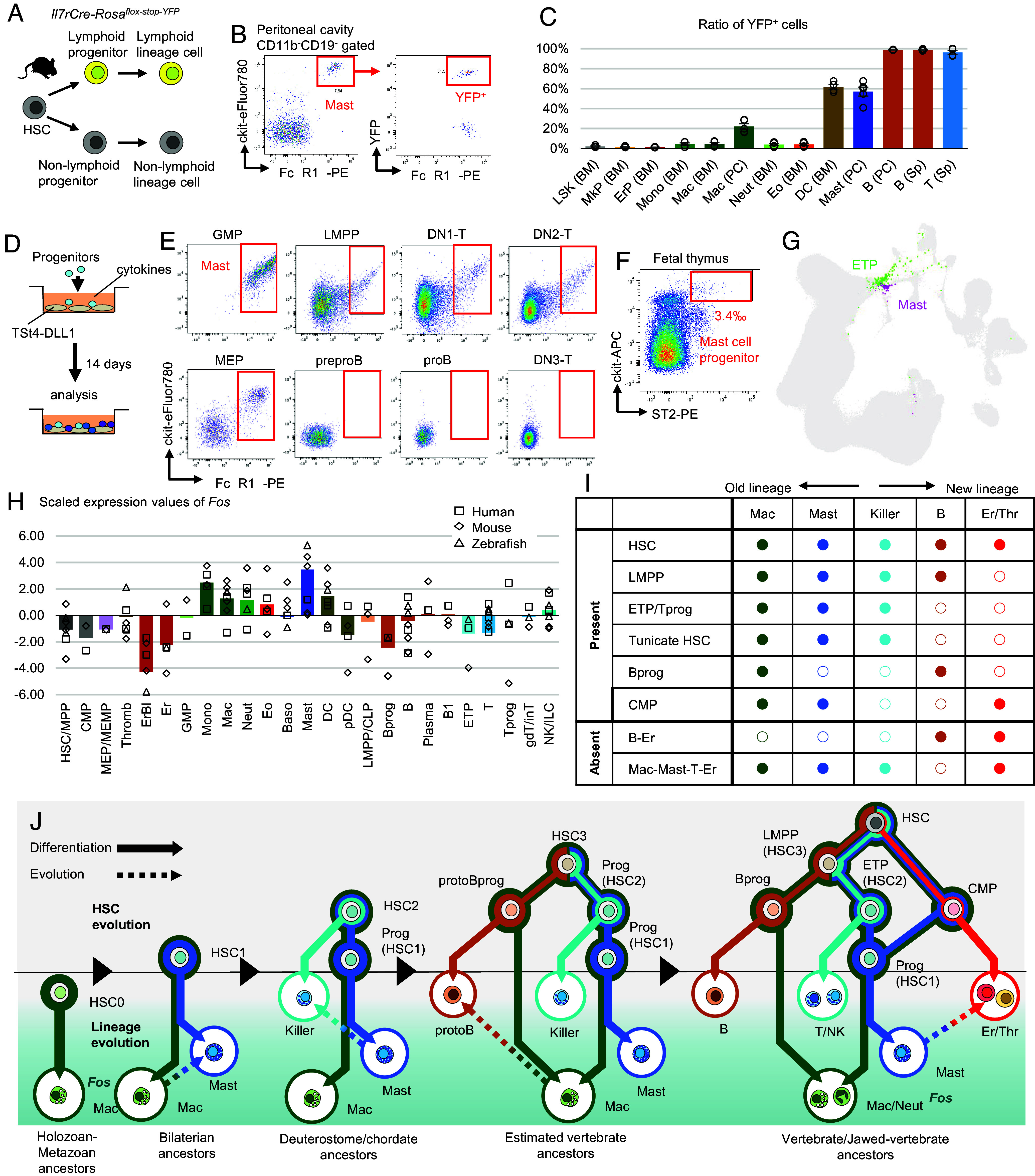
Retention of macrophage and mast cell potential in hematopoietic differentiation pathways as a vestige of the evolutionary history. (*A*) Lymphoid progenitors and their progeny are marked with YFP in *Il7r-Cre; Rosa^flox-stop-YFP^* mice. (*B*) Representative flow cytometric profiles of YFP^+^ gating in mast cells (n = 5). (*C*) Percentage of YFP^+^ cells in *Il7r-Cre; Rosa^flox-stop-YFP^* mice. Mean values ± SE of biological replicates (n = 4 to 5). (*D*) Schematic illustration of the in vitro culture system for evaluating mast cell potential. Various hematopoietic progenitors were cocultured with TSt4-DLL1 stromal cells in the presence of cytokines. After 14 d of culture, the generated cells were evaluated by flow cytometry. (*E*) Flow cytometric profiles of generated cells from each progenitor in vitro. FcεR1α^+^c-kit^+^ mast cells were gated. Representative data of biological replicates (n = 3). (*F*) Flow cytometric profiles of E16.5 fetal thymus gated on Lin^−^CD25^−^ cells. Representative data of biological replicates (n = 3). (*G*) Human thymus single-cell RNA-seq data in public databases showed that mast cells differentiated from ETPs in the thymus (Human Cell Atlas Developmental, https://app.cellatlas.io/thymus-development/dataset/8/scatterplot) (54). (*H*) Expression levels [log2(TPM+1), differences from mean values] of *Fos* in vertebrate blood cell lineages. (*I*) Table of multi/oligo-lineage progenitors and their lineage potentials that are already known and newly found in this study. There are no B/Er or Mac/mast/T/Er progenitors. (*J*) Schematic illustration of the reciprocal relationship between evolution and differentiation of blood cell lineages. Solid and broken lines indicate directions of differentiation and evolution, respectively. BM, bone marrow; Bprog, B-cell progenitor; Er, erythrocyte; GMP, granulocyte-monocyte progenitor; Mac, macrophage; Neut, neutrophil; PC, peritoneal cavity; protoB, prototypic B cell; Sp, spleen.

To verify this hypothesis, we first performed an in vivo assay and examined whether lymphoid progenitors differentiated into mast cells, using *Il7r-Cre; Rosa^flox-stop-YFP^* mice whose lymphoid lineage cells are marked with YFP. We observed that about half of the mast cells in the peritoneal cavity were marked with YFP, which was remarkably higher than the frequency of other non-lymphoid cells such as neutrophils or erythroid cells (Fig. 4 *A*–*C* and *SI Appendix*, Fig. S8). This indicated that about half of the mast cells were derived from lymphoid progenitors. *Il7r-Cre; Rosa^flox-stop-YFP^* mice also showed that about 20% of macrophages in peritoneal cavity was derived from lymphoid cells (Fig. 4*C*), which was consistent with previous reports that B-1 cells in the peritoneal cavity have potential to differentiate to macrophages (50, 51). In vitro experiments also demonstrated that mast cells derived from LMPPs and double negative (DN)1-2 T cell progenitors, indicating their mast-cell potential (Fig. 4 *D* and *E*). We also detected mast-cell potential in megakaryocyte-erythrocyte progenitors (MEPs), in line with previous reports (48, 52, 53), but not in B-cell progenitors (Fig. 4*E*). We further detected mast cell progenitors in mouse fetal thymus (Fig. 4*F*). Single-cell RNA-seq data of human thymocytes, from a previous report, also showed the existence of mast cells within the thymus and a close differentiation pathway between early T-cell precursors (ETPs) and mast cells (Fig. 4*G*) (54). All these data indicate that T-cell progenitors, as well as MEPs, can differentiate into mast cells. In addition to mast cell potential, we previously reported that macrophage potential is retained in almost all the blood lineage progenitors (9, 55). Consequently, macrophages and mast cells—the first two lineages to emerge in the evolutionary history of blood cells—are also the two lineages whose differentiation potentials are most widely retained in modern hematopoiesis. This broad retention can be interpreted as a vestige of their evolutionary history. We further investigated the underlying reasons for this widespread retention of evolutionarily ancient cell lineage potential in hematopoietic differentiation. Notably, among vertebrate blood cell lineages, both macrophages and mast cells exhibited high expression of *Fos* (Fig. 4*H*), a TF identified as crucial for the initial blood cells of early animals (Fig. 1*D* and *SI Appendix*, Fig. S3). Therefore, we considered that differentiation potentials of evolutionary old cell lineages that have more profoundly inherited an ancestral genetic program were widely retained in developmental pathways. Next, we verified our findings about differentiation potentials analyzing chromatin accessibilities with public murine ATAC-seq data (*SI Appendix*, Fig. S9*A*) (56–58). In line with the widest differentiation potential of macrophages, *Cebpa* locus was open at an early differentiation stage of HSCs as well as oligo-lineage progenitors, such as CLPs and DN1-2 cells. On the other hand, loci of *Bcl11b* and *Pax5*, which are specific TFs for T cells and B cells, respectively, were closed at HSCs, and got open around later stages. In addition, locus of *Fos*, which is one of key TFs determining blood cells from an ancient holozoan ancestor, was open throughout all the differentiation stages and lineages. Furthermore, when TF homologs were compared in detail, loci of mast-cell-related TFs, such as *Gata2* and *Tcf7l1* were open from early stages, while those of T-cell-related TFs, such as *Gata3* and *Tcf7* were open around later stages. These data are compatible with why mast-cell potential was more widely retained compared to T-cell potential. ATAC-seq data also suggested close relationship between mast, T/NK, and erythrocyte lineages and also relationship between macrophage and B lineages. When *Cdc42* gene which was constitutively highly expressed in various blood cell lineages was investigated, we found one candidate of regulatory elements at the first intron which was opened in monocytes, macrophages, neutrophils, DCs, and B cells, but closed in T cells, NK cells, and ILC2 (*SI Appendix*, Fig. S9*A*). We also focused on regulatory elements in the first intron of the *Vav1* gene which was almost specifically expressed in blood cells (59). One region was open in macrophage lineage cells and B cells, and another adjacent region was open in mast cells, T/NK/ILCs, and MEPs, respectively. These regions contain possible binding sites of FOS, CEBPα/β/δ/ε, and PU.1 in the macrophage/B elements, and those of RBPJ, TCF7, MEIS, GATA, RUNX in the mast/T/NK/erythrocyte element, respectively (*SI Appendix*, Fig. S7*D*). These shared regulatory elements support the possibility that T/NK cells and erythrocytes evolved from ancestral mast cells, and B cells evolved from ancestral macrophages. We also evaluated similarities of chromatin accessibilities throughout whole genes, and this comprehensive analysis showed that B cells were more similar to macrophages and DCs than to T cells, supporting the close relationship between macrophages and B cells (*SI Appendix*, Fig. S10).

Here, we further discuss the relationship between the evolutionary trajectory of blood cells and the developmental process in vertebrate hematopoiesis. Our findings suggest that the evolutionary history of blood cell lineages is reciprocally mirrored in the differentiation process of hematopoiesis, implying that lineage restriction during development largely reverses the lineage acquisition sequence observed in evolution (Fig. 4 *I* and *J*). It is well known that HSCs differentiate into multi- and oligo-lineage progenitors such as CMP/MEMPs (48, 60, 61), or LMPPs and ETPs (55, 60, 62). Results of this study suggest that multi/oligo-lineage progenitors have the potential of some lineages and their ancestral lineages (Fig. 4*I*). For example, DN1 T-cell progenitors or ETPs possess T-cell, NK-cell, mast-cell, and macrophage potentials, reflecting that T cells, NK cells, and mast cells evolved from prototypic mast cells that had previously evolved from macrophages (Fig. 4*I*). CMPs have erythrocyte, thrombocytes, mast-cell, and macrophage potentials, reflecting that erythrocytes and thrombocytes evolved from ancestral mast cells (Fig. 4*I*). On the other hand, B-erythrocyte progenitors do not exist because they are in phylogenetically distant clades (Fig. 4*I*). It is also suggested that old HSCs were inherited as oligo-lineage progenitors when ancestors acquired novel lineages. For example, deuterostome ancestors are estimated to have had HSCs with potentials of macrophage and mast/killer lineages as tunicate HSCs do. These old HSCs are inherited as ETPs in current vertebrates which also retain macrophage and mast/killer lineage potentials. If it is a general law that the lineage acquisition process is reciprocal to the developmental process, a more detailed evolutionary history of vertebrate-unique lineages can be estimated, and B lineage acquisition are considered to have preceded erythrocyte/thrombocyte lineage acquisition (Fig. 4*J*). If the erythrocyte/thrombocyte lineage had emerged prior to the B lineage, we would expect to find progenitors with T-cell, NK-cell, mast-cell, erythrocyte, thrombocyte, and macrophage potentials, but such progenitors are not observed (Fig. 4*I*).

## Discussion

The availability of diverse species’ genomes has significantly advanced our understanding of interspecies similarities, differences, and the phylogeny of organisms (63–66). Yet, the evolutionary history of individual cell lineages remains to be further explored. Because cell lineage identity is a product of gene expression, just genomic data alone cannot explain how specialized lineages emerged. Our study addresses this gap with regards the metazoan blood cell lineages by directly comparing gene expression profiles across a broad spectrum of metazoan species and their unicellular relatives, thereby predicting the detailed evolutionary history of metazoan blood cell lineages.

We propose that the dawn of Metazoa was not merely a transition to multicellularity but also the beginning of a new defensive system, centered on macrophage-like phagocytes. When the first multicellular metazoans evolved from their unicellular ancestors, they evolved non-blood cells, such as epithelial cells. At the same time, the need for internal defense against ubiquitous pathogens like bacteria and viruses gave rise to the initial blood cells: macrophage-like phagocytes. Our work shows that these initial blood cells inherited and repurposed the genetic programs of their unicellular progenitors (Fig. 1) (67). Our data suggest that this repurposing was mediated by *Fos*. Of note, *Fos* homologs were detected in metazoan species, choanoflagellate *S. rosetta*, and *C. owczarzaki*, but not in non-holozoan species, such as yeast, in this study (Dataset S5). Therefore, acquisition of *Fos* and *Fos*-mediated blood cell programs was one of the key events which enabled holozoan ancestors to evolve toward multicellular animals with blood cells. As evolution progressed, the rise of animal-on-animal parasitism created a new selective pressure. In response to this new selective pressure, bilaterian ancestors diversified their defense system, acquiring prototypic mast cells (Fig. 2). These cells, containing granzyme-like proteases, appear to have been primarily specialized for antiparasitic defense, marking the first major divergence within the blood cell lineages. The granular or crystalline inclusions observed in T/NK cells, mast cells, tunicate morula and test cells, sea urchin CLSs, and fly CCs further suggested that the prototypic mast cells also contained granules. While these granules likely contained cytotoxic molecules such as granzyme homologs, the mechanism of engagement against parasites differed from the direct killing characteristic of T cells and NK cells. Unlike T/NK cells, which deliver granzymes into target cells via perforins, tunicates, sea urchins, and flies lack perforin homologs (*SI Appendix*, Fig. S6*G*), and mammalian mast cells do not express perforin. Instead, we propose that the prototypic mast cells may have combated parasites through perforin-independent granzyme action or by modulating the immune microenvironment as current mast cells do (68, 69). The subsequent evolutionary trajectory in deuterostome and vertebrate ancestors witnessed further diversification (Fig. 2). The ancestors developed T/NK cells from these ancestral mast cells, with their functional proteins diversifying into specialized granzymes and acquiring perforin. Concurrently, vertebrates acquired other novel lineages, notably erythrocytes and B cells. Our analyses consistently predict that the erythrocyte/thrombocyte lineage evolved from the mast cell branch, while the B lineage emerged from the macrophage branch. They also developed hematopoietic organ for mast/killer lineages at the gill edges, the origin of thymus (Fig. 3). This evolutionary history is exposed on the current hematopoietic system as a reciprocal lineage restriction process (Fig. 4).

While this study offers a comprehensive framework for the evolutionary history of blood cell lineages, some limitations warrant discussion. First, it is hard to estimate the emergence of acquired immunity characterized by diversified antigen receptors formed by the RAG-mediated rearrangement of gene segments in T cells and B cells. There is also the possibility that acquired immunity preceded erythrocyte emergence and vice versa. Although, a reciprocal relationship between evolution and differentiation suggests that the B-cell lineage preceded the erythrocyte lineage (Fig. 4 *I* and *J*), the prototypic B cells may have performed just antigen-presentation without diversified receptors. Investigations utilizing other species such as lamprey which have variable receptors on their lymphocytes as well as erythrocytes (70–72) can address this question. It was recently reported that lamprey’s variable lymphocyte receptors are mediated by cytidine deaminase 2 (73). Although a slight possibility remains that lamprey secondarily lost RAG-mediated acquired immunity, it suggests that the acquired immunity emerged after the bifurcation of jawed and jawless vertebrates and followed the emergence of erythrocytes. When we expand species toward Protostomia, some deep sea shells were reported to have several blood cell lineages (74). Investigating these mollusk blood cells can also tell us information about history of blood cell lineage diversification, even if they are less related to acquired immunity. As a second limitation, there may be other TFs important in ancestral cell lineages but not identified in this study. This study succeeded in finding key TFs in ancestral cell lineages such as *Fos* (Fig. 1*D* and *SI Appendix*, Fig. S3), but this does not suggest that they are whole TFs determining blood cells in ancestors, and some additional ones can be identified in the future by investigating other animals or unicellular organisms. Third, estimated TF bindings sites were not validated by ATAC-seq or immunoprecipitation experiments in tunicate or sea urchin, and future investigations are required to confirm the TF networks estimated in this study. Accumulation of transcriptome and epigenetic data of more animal species and progress in analytic methods including homolog estimation are required to reconstruct more detailed and comprehensive phylogeny of cell lineages.

In conclusion, our study provides the phylogeny of animal blood cells based on transcriptional similarities, revealing that the complex vertebrate immune system is a highly specialized expansion of an ancient premetazoan toolkit successfully scaled to the higher demands of animal multicellularity. Our framework explains the deep continuity between ancestral unicellular organisms to sponge archaeocytes and mammalian macrophages, while providing a robust lens to interpret the functional vestiges preserved in modern animal development.

## Materials and Methods

Full detailed Materials and Methods are in *SI Appendix*.

### Mice.

*Il7r-Cre; Rosa26^flox-stop-YFP^* mice were generated from *Il7r-Cre* mice (75) and *Rosa26^flox-stop-YFP^* (76) mice, and maintained in our animal facility. Proportion of YFP expressing cells in each blood cell lineage was examined. In vitro experiments about mast cell differentiation potential were performed with progenitors isolated from wild type mice. All mice were maintained under SPF conditions. All experiments were performed in accordance with the guidelines of the Kyoto University Animal Experiment Committee and approved by our institutional committee (K-23-29 and K-24-31-2).

### Tunicate and Sea Urchin.

*Ciona robusta* juveniles and adults were obtained from the National BioResource Project for *Ciona*. *Hemicentrotus pulcherrimus* adults were obtained from the Misaki coast with permission. RNA sequencing of blood cells and WISH were performed.

### Capsaspora owczarzaki.

*C. owczarzaki* was cultured and maintained as previously described (77, 78). Functions of *Fos* and *Myb* homologs in *C. owczarzaki* were examined by enforced expression establishing knock-in cells.

### Cross-Species Transcriptomic Comparison.

For the cross-species comparison, we strategically selected species that represent the key nodes of animal evolution (Fig. 1*A*). Then, we identified homologs in the proteome data using OrthoFinder (79). We then normalized TPM values only for the subset of conserved Orthogroups among analyzed species and transformed them to log2 (TPM +1). Standardized TPM values by calculating differences from mean values among each dataset were analyzed, and phylogenetic trees were constructed based on Pearson’s correlation values. Differentially expressed Orthogroups and KEGG pathways were also analyzed (*SI Appendix*). To complement transcriptomic analysis, TF binding site estimation and chromatin accessibility analysis were also performed (*SI Appendix*).

## Supplementary Material

Appendix 01 (PDF)

Dataset S01 (XLSX)

Dataset S02 (XLSX)

Dataset S03 (XLSX)

Dataset S04 (TXT)

Dataset S05 (TSV)

Dataset S06 (TSV)

Dataset S07 (XLSX)

## Data Availability

RNA-seq data of tunicate and sea urchin newly obtained for this study are available at the DNA Data Bank of Japan (DDBJ) database [PRJDB16579/E-GEAD-1216 (80, 81) and PRJDB17706/E-GEAD-1217 (82, 83)]. Representative codes used in this study are in Datasets S1 and S4.
