# Supplementary material for: Animals have expanded the evolutionary legacy of unicellular ancestors in blood cells
Source: Proc Natl Acad Sci U S A. 2026 May 28;123(23):e2528110123. doi: 10.1073/pnas.2528110123 (PMC13250551; doi:10.1073/pnas.2528110123)
Supplement: Supplementary file 1 — Appendix 01 (PDF) [file pnas.2528110123.sapp.pdf]

PNAS

Supporting Information for
Animals have expanded the evolutionary legacy of unicellular ancestors in blood cells

Yosuke Nagahata[a,b,c]*, Yuji Nishimura[a], Ryota Kaitani[a], Jason Cheok Kuan Leong[d], Izumi Oda-Ishii[e], Hisanori Kohtsuka[f], Shinya Abe[g,h], Tasuku Ishida[e], Marina Carmona-Rivas[b], Sebastian R. Najle[i], Elena Casacuberta[b], Koichi Ikuta[g], Toru Miura[f], Michio Ogasawara[j], Naoki Irie[d], Yutaka Satou[e], Iñaki Ruiz-Trillo[b,k]*, Hiroshi Kawamoto[a]*

*Corresponding authors: Yosuke Nagahata, Iñaki Ruiz-Trillo, and Hiroshi Kawamoto
Email: yosuke.nagahata@gmail.com
inaki.ruiz@ibe.upf-csic.es
kawamoto@infront.kyoto-u.ac.jpx

This PDF file includes:

Supporting text
Figures S1 to S12
Legends for Datasets S1 to S7
SI References

Other supporting materials for this manuscript include the following:

Datasets S1 to S7

Supporting Information Text

Materials and Methods

Data and code availability

For comparison of gene expression profiles among different species, public data of human, mouse, zebrafish, tunicate, fly, nematode, sponge, and several unicellular organisms were analyzed (*SI Appendix*, Dataset S1) (53, 56, 84-91). RNA-seq data of tunicate and sea urchin newly obtained for this study are available at the DNA Data Bank of Japan (DDBJ) database (PRJDB16579/E-GEAD-1216 and PRJDB17706/E-GEAD-1217). Sequenced reads of tunicate were mapped on the Ensembl model of the *C. robusta* genome, and those of sea urchin were mapped on the *H. pulcherrimus* genome (92). For searching WISH data of genes highly expressed in morula cells, TPM values mapped on the KY genome model of C. robusta were analyzed. Representative codes used in this study are in *SI Appendix*, Dataset S4. All the other data are available in the manuscript or the Supplemental data, and further information is available upon request.

Mice

Il7r-Cre; Rosa26$^{flox\text{-}stop\text{-}YFP}$ mice were generated from *Il7r-Cre* mice (75) and *Rosa26*$^{flox\text{-}stop\text{-}YFP}$ (76) mice, and maintained in our animal facility. All mice were maintained under SPF conditions. All experiments were performed in accordance with the guidelines of the Kyoto University Animal Experiment Committee and approved by our institutional committee (K-23-29 and K-24-31-2). We used mice 4 months old or younger and did not distinguish sex because we did not observe sex differences in preliminary experiments.

Tunicate

Ciona robusta (also Called *Ciona intestinalis*, type A) juveniles and adults were obtained from the National BioResource Project for *Ciona*.

Sea urchin

Hemicentrotus pulcherrimus adults were obtained from the Misaki coast with permission.

Tunicate blood cells

The blood of *C. robusta* was aspirated by cardiac puncture with a syringe in the presence of artificial seawater (Tomita Pharmaceutical) with 10mM EDTA. Blood cells were collected from pooled blood of several dozen tunicates by centrifugation (800 g, 5 minutes, 4℃). To distinguish phagocytes, blood cells were incubated with pHrodo-green zymosan beads in the artificial seawater for 1 hour at room temperature. Isolation of each lineage of blood cells was performed by FACS. Hemoblasts, also called lymphocyte-like cells, were identified as small-size cells, and large-size cells were subdivided into 3 fractions: macrophages as pHrodo-green positive cells, morula cells/granulocytes with basophilic granules as autofluorescence-positive cells detected by a violet laser (405 nm) and V450 channel (450 nm), and progenitors (other cells) as pHrodo-green negative, autofluorescence-negative cells. Cells with autofluorescence detected in the APC channel (640nm laser and 670nm filter) were sorted as amoebocytes (also called hyaline amoebocytes), as previously reported (13), and they showed a transcriptome profile similar to macrophages. Test cells were collected by removing chorion from eggs with 1% sodium thioglycolate + 0.05% actinase E. Some test cells were autofluorescent as detected in both FITC (488nm laser and 530 nm filter) and PI channels (488nm laser and 695 nm filter). Because these autofluorescence-positive and -negative test cells showed similar transcriptome profiles, we analyzed RNA-seq data of test cells without autofluorescence-based classification. The morphology of the cells was observed by microscopy (BIOREVO BZ-9000, KEYENCE).

Sea urchin blood cells (coelomocytes)

The blood (coelomic fluid) of *H. pulcherrimus* was aspirated by puncture with a syringe in the presence of artificial seawater (Tomita Pharmaceutical). Coelomocytes from 2 individuals were pooled and analyzed. Amoebocytes, vibratile cells, CLSs, and red cells were separated through an iodixanol discontinuous gradient (22). Because gene expression profiles of the two replicate samples of vibratile cells differed, they were excluded from further analyses. In the cross-species comparison, we used only amoebocytes and CLSs, excluding red cells because the function of red cells is not well known. The morphology of the cells was observed by microscopy (BIOREVO BZ-9000, KEYENCE).

Reanalysis of vertebrate RNA-seq data

To obtain enough blood cell RNA-seq data covering a wide range of cell lineages, we reanalyzed some single-cell RNA-seq data and generated virtual cell data with sum count values for each lineage of cells (*SI Appendix*, Dataset S1: Hs3, Mm4, Mm5, Dr1, Dr2, Dr3, Dm1, and Ce). Although we essentially used the original cell annotations, we refined the cell annotations for human blood cells (Hs3) (53), mouse fetal blood cells (Mm4) (86), and zebrafish cells (Dr2) (86) with the Leiden method (igraph 0.11.4 and leidenalg 0.10.2) and expression levels of some representative genes using scanpy 1.10.1. For the human blood cell dataset, eosinophils/basophils and mast cells were re-clustered (n_neighbors=30, n_pcs=50, resolution=0.5) (9 clusters; eosinophil/basophil, cluster #4, 6, 7; mast cell, #0-3, 5, 8) (*SI Appendix*, Fig. S11A, Hs3). Because some mast cells in single cell datasets barely expressed *FCER1A*/*Fcer1a*, a prototypic mast cell marker, they were regarded as mast cell progenitors. For the mouse fetal blood cell dataset (*SI Appendix*, Fig. S11B, Mm4), macrophages and EMPs were re-clustered (n_neighbors=20, n_pcs=50, resolution=1.2) (15 clusters; EMP, cluster #11; macrophage, #0, 2-5, 8, 10; basophil, #13; eosinophil #14; mast cell progenitor-1, #7; mast cell progenitor-2, #1, 12). Cluster 6, 7, and 9 were further clustered with resolution=4.0 (B cell, #9-9; mast cell, #9-32; DC, #6-24; ILC, #6-29; macrophage, #6-non24/29, #7-non15). In the zebrafish dataset (*SI Appendix*, Fig. 11C, Dr2), we refined the annotation as described in *SI Appendix*, Dataset S2. In particular, we reanalyzed the original data for eosinophils, basophils, and mast cells. Because zebrafish genes highly homologous to *Fcer1a* or *Il1rl1* were not identified, at first, we tried to find genes characteristic of eosinophils/basophils/mast cells. We focused on granzymes and MCPTs, which are highly expressed in those lineages. We analyzed amino acid sequences of OG0000005 containing granzymes and MCPTs and found that complement factor D (CFD) protein, encoded by the *cfd* gene, was similar to granzymes and MCPTs (*SI Appendix*, Fig. S4I). Indeed, the human protein database shows that CFD is expressed in mast cells (https://www.proteinatlas.org/ENSG00000197766-CFD/tissue+cell+type). Thus, we regarded zebrafish CFD as a CFD/granzyme/MCPT homolog and examined its expression levels. In kidney marrow data, cells originally annotated as eosinophils and highly expressing *cfd* were annotated as basophils because they expressed *cebpa* and *gata2a* but not *gata1a/b*. In the thymus data, cells of cluster 31, originally annotated as epithelial cells, expressed *epcam*, *cd44a*, *cfd*, and *gata2a*. Because mast cells can express EPCAM protein (93), we regarded them as mast cell-like cells after excluding *bcl11ba*+ nurse cell-like cells (n_neighbors=10, n_pcs=50, resolution=0.5). Cell lineage annotations for UMAP and phylogenetic tree analysis are described in *SI Appendix*, Dataset S2.

Reanalysis of fly blood cell RNA-seq data

Fly blood cell single cell RNA-seq data (90) were used for this analysis, and data from the 10X method were selected. For each lineage, only differentiated cells were included: PM1 and PM11 for macrophage-like plasmatocytes, PM4, PM5, and PM6 for antimicrobial peptide-expressing plasmatocytes, LM2 for lamellocytes, and CC1 and CC2 for crystal cells.

Sponge RNA-seq data

RNA-seq data from sponge cells (91) were used for this analysis, and mean values of archaeocytes, choanocytes, and pinacocytes were calculated to compare cell lineages with other species. In analysis of blood cell lineages, archaeocytes were grouped into two as phagocytic macrophages and non-phagocytic normal/stem-cell-like archaeocytes based on expression levels of phagocytosis-related genes.

Cross-species transcriptomic comparison

For the cross-species comparison, we first identified homologs in the proteomes data. For analysis of whole body cell lineages, proteomes of *Homo sapiens*, *Mus musculus*, *Danio rerio*, *Ciona robusta*, *Drosophila melanogaster*, *Caenorhabditis elegans*, *Amphimedon queenslandica*, *Salpingoeca rosetta*, *Capsaspora owczarzaki*, *Ministeria vibrans*, *Creolimax fragrantissima*, *Saccharomyces cerevisiae*, *Dictyostelium discoideum*, *Chlamydomonas reinhardtii*, *Thalassiosira pseudonana*, *Prometheoarchaeum syntrophicum*, *Haloferax volcanii*, *Escherichia coli*, and *Staphylococcus aureus* were compared, and homologs were identified using the OrthoFinder (version 2.5.5) (*SI Appendix*, Fig. S1A) (79). Instead of the default algorithm of Diamond, we used MMseqs2 (94) and a modified MCL inflation parameter (I = 1.3) for identifying homologs. For analysis blood cell lineages, *Homo sapiens*, *Mus musculus*, *Danio rerio*, *Ciona robusta*,

Branchiostoma lanceolatum, *Strongylocentrotus purpuratus*, *Hemicentrotus pulcherrimus*, *Acanthaster planci*, *Octopus bimaculoides*, *Drosophila melanogaster*, *Nematostella vectensis*, *Trichoplax adhaerens*, *Mnemiopsis leidyi*, *Amphimedon queenslandica*, and *Capsaspora owczarzaki* with a higher MCL inflation parameter (I = 1.8) (79). Amino acid sequences were obtained from Ensembl and other databases and previous reports (*SI Appendix*, Dataset S2) (77, 78, 91, 92, 95-97). Some amino acid sequences were corrected as previously described (8). For the *Cebpa/b/d/e* homolog of *C. owczarzaki* (*CAOG_02294*), sequences in the NCBI database were used. ENSCING00000009562 of tunicate was divided into two different proteins (ENSCING00000009562e and ENSCING00000009562r) because it consists of Ring1a/b and an exosome component protein in the ghost database. In comparison of blood cell lineages, we additionally revised some homolog groups of TFs. *Cebpa/b/d/e* homologs were distinguished from *Cebpg* homologs based on a previous report (8). *Spib* homologs were manually added to *Spi1/Spic* homologs. Zebrafish *Cebp1* and *C. owczarzaki CAOG_03823* were manually added to *Cebpa/b/d/e* homologs and *Cebpg* homologs, respectively. *Ikzf1-4* homologs were separated from the original large homolog group (OG0000000) based on analysis with a higher inflation parameter (I = 2.0). Lists of genes and Orthogroups are in *SI Appendix* Dataset S5 and S6. If one Orthogroup contained two or more homologs in one species, the total TPM values of the homologs were used for the TPM value of the Orthogroup. We then normalized TPM values only for the subset of conserved Orthogroups among analyzed species and transformed them to log2 (TPM +1). For analysis containing unicellular organisms, number of Orthogroups shared among animals and unicellular organisms were too small, and we selected Orthogroups shared among animals: human, mouse, zebrafish, tunicate, fly, nematode, and sponge. To minimize batch effects and inter-species differences, we standardized TPM values by calculating differences from mean values among each dataset with a standard scaler (centered with 0 and not divided by SD). For unicellular organisms, scaling was performed among whole unicellular species instead of scaling among each species.

Genome-wide analysis of chromatin accessibilities

Open chromatin region (OCR) values in a previous report (56) were re-analyzed. Because original OCR values were converted back to linear scale from log-transformed and quantile-normalized values, and distributed from minimum values of approximately 1, OCR-1 values were calculated. These modified OCR values were further utilized and analyzed in the same way as TPM values in transcriptome data.

Phylogenetic trees

Pearson's correlation values were calculated based on the scaled log2 (TPM+1) values, and these values were scaled with a minimax scaler (minimum=0 and max=1): the furthest correlation value was 0 and the nearest correlation value (value between identical samples) was 1. We selected Pearson's correlation values based on data distribution normality evaluated by histograms (*SI Appendix*, Fig. S12). For analysis of whole body cell lineages based on 25 TFs, more than half of cell lineages showed normality with Shapiro-Wilk normality test, Pearson's correlation values were selected (*SI Appendix*, Dataset S7). Distance values were calculated by 1 - (correlation values) and phylogenetic trees were drawn with the distance values and neighbor-joining method of DendroPy 4.6.1 (98). For the phylogenetic trees including non-blood cells, Spearman's correlation values were also calculated.

We excluded noisy genes as follows. For the trees including blood cells, non-blood cells, sponge and unicellular organisms, we excluded cell cycle related Orthogroups and non-variable Orthogroups with less than 1 maximum TPM values or 0.25 of SD values in human, tunicate, fly, nematode, and sponge data. SD values were calculated with log2(TPM+1) values. Then, correlation values were calculated based on expression matrix of remained 1610 Orthogroups. For the tree including blood cells, non-blood cells, sponge and unicellular organisms and selecting TFs, highly variable TF Orthogroups with more than 15 TPM values and 0.5 SD values in human, mouse, zebrafish, fly, and sponge data were further selected, and correlation values were calculated based on remained 25 Orthogroups. For the tree of vertebrate, tunicate, and sea urchin blood cells, cell cycle related Orthogroups and non-variable Orthogroups with less than 15 maximum TPM values or 0.6 of SD values in human (Hs3), mouse (Mm1), zebrafish (Dr2) and tunicate data were excluded and remained 80 TF Orthogroups were analyzed. For the tree of vertebrate, tunicate, sea urchin, and fly blood cells, cell cycle related Orthogroups and non-

variable Orthogroups with less than 1 maximum TPM values or 0.5 of SD values in human (Hs3), mouse (Mm1), zebrafish (Dr2), and tunicate data were excluded and remained100 TF Orthogroups were analyzed. For the tree of blood cell lineages and cell stages of *C. owczarzaki*, non-variable Orthogroups with less than 1 maximum TPM values or 0.4 of SD values in mouse (Mm1), tunicate, sponge, and *C. owczarzaki* data were excluded and remained 37 TF Orthogroups were analyzed. For the tree based on chromatin accessibilities at a gene level, genes with SD values higher than 1.5 and with OCR values higher than 15 in more than half cells were selected. For the tree based on chromatin accessibilities at an Orthogroup level, Orthogroups with SD values higher than 1.5 and with OCR values higher than 31 in more than half cells were selected.

Multiscale bootstrapping

To evaluate reliability of tree nodes, bootstrap resampling was performed with 100 replicates, and we calculated AU (approximately unbiased) values with multiscale bootstrapping (99, 100). For the trees including blood cells, non-blood cells, sponge and unicellular organisms based on 1610 Orthogroups (N = 1610), 100 bootstrapped expression matrixes with following Orthogroup numbers were generated: 644 (0.4N), 805 (0.5N), 966 (0.6N), 1127 (0.7N), 1288 (0.8N), 1610 (1.0N), 1932 (1.2N), 2254 (1.4N), 2737(1.7N), 3220(2.0N), and 4025 (2.5N). For the tree including blood cells, non-blood cells, sponge and unicellular organisms and selecting TFs based on 25 Orthogroups (N = 25), 1000 bootstrapped expression matrixes with following Orthogroup numbers were generated: 5 (0.2N), 6 (0.25N), 8 (0.3N), 10 (0.4N), 13 (0.5N), 15 (0.6N), 20 (0.8N), 25 (1.0N), 50 (2.0N),100 (4.0N), 150 (6.0N), 200 (8.0N), 250 (10N), 300 (12N), 250 (14N), 400 (16N), and 500 (20N). For the tree of vertebrate, tunicate, and sea urchin blood cells based on 80 Orthogroups (N = 80), 1000 bootstrapped expression matrixes with following Orthogroup numbers were generated: 20 (0.25N), 24 (0.3N), 32 (0.4N), 40 (0.5N), 48 (0.6N), 56 (0.7N), 64 (0.8N), 80 (1.0N), 96 (1.2N), 112 (1.4N), 136 (1.7N), 160 (2.0N), 200 (2.5N), 240 (3.0N), and 320 (4.0N). For the tree of vertebrate, tunicate, sea urchin, and fly blood cells based on 100 Orthogroups (N = 100), 1000 bootstrapped expression matrixes with following Orthogroup numbers were generated: 25 (0.25N), 30 (0.3N), 40 (0.4N), 50 (0.5N), 60 (0.6N), 70 (0.7N), 80 (0.8N), 100 (1.0N), 120 (1.2N), 140 (1.4N), 170 (1.7N), 200 (2.0N), 250 (2.5N), 300 (3.0N), and 400 (4.0N). For the tree of vertebrate, tunicate, fly, and sponge blood cells and *C. owczarzaki*, based on 37 Orthogroups (N = 37), 1000 bootstrapped expression matrixes with following Orthogroup numbers were generated: 9 (0.25N), 11 (0.3N), 15 (0.4N), 19 (0.5N), 22 (0.6N), 26 (0.7N), 30 (0.8N), 37 (1.0N), 44 (1.2N), 52 (1.4N), 63 (1.7N), 74 (2.0N), 93 (2.5N), 111 (3.0N), and 148 (4.0N). For the tree based on chromatin accessibilities of 1236 genes (N = 1236), 100 bootstrapped OCR matrixes with following gene numbers were generated: 618 (0.5N), 742 (0.6N), 865 (0.7N), 989 (0.8N), 1236 (1.0N), 1483 (1.2N), 1730 (1.4N), 2101 (1.7N), and 2472 (2.0N). For the tree based on chromatin accessibilities of 545 Orthogroups (N = 545), 100 bootstrapped OCR matrixes with following gene numbers were generated: 273 (0.5N), 327 (0.6N), 382 (0.7N), 436 (0.8N), 545 (1.0N), 654 (1.2N), 763 (1.4N), 927 (1.7N), and 1090 (2.0N).

TFs and cell cycle related genes

For selecting TFs, we used the AmiGO2 database (http://amigo.geneontology.org/amigo). TF candidates were searched with the words “transcription factor” in *M. musculus*, and 1712 genes were identified. Among them, 485 non-TF genes, such as polycomb proteins, were manually removed, and the remaining 1227 genes were selected as TFs (*SI Appendix*, Dataset S3). For cell cycle related genes 1064 cell cycle related genes were selected also using AmiGO2 database excluding words of “regulating”.

Differentially expressed Orthogroups

In analysis of whole body cell lineages, we widely selected differentially expressed Orthogroups in each cell lineage as Orthogroups with > 0 or < 0 scaled values. Orthogroups differentially expressed in whole blood/leukocytes were regarded as differentially expressed Orthogroups in blood cells. We then selected differentially expressed Orthogroups shared among different species. Orthogroups differentially expressed commonly among Vertebrata (human, mouse, and zebrafish), Deuterostomia (human, mouse, zebrafish, and tunicate), Bilateria (human, mouse, zebrafish, and 2 of tunicate, fly and nematode), and Metazoa (human, mouse, zebrafish, sponge and 2 of tunicate, fly and nematode) were selected for vertebrate, deuterostome, bilaterian, and metazoan ancestor blood cells, respectively.

In analysis about blood cell lineages, differentially expressed Orthogroups were defined as Orthogroups with >$2^{0.5}$ scaled values, and we focused on highly expressed Orthogroups. If a lineage contained two or more cells, Orthogroups highly expressed in either of the cells were regarded as differentially expressed Orthogroups of the lineage. For example, Orthogroups differentially expressed in either of monocytes, macrophages, and neutrophils, were selected for the vertebrate macrophage lineage. In fly blood cells, we selected CCs as mast/killer lineage cells and because only CCs highly expressed granzyme homologs. As for vertebrates, Orthogroups highly expressed in all of human, mouse, and zebrafish were selected. In the analysis of animal blood cell lineages and three stages of *C. owczarzaki*, Orthogroups differentially expressed in HSCs compared to macrophage lineages, and Orthogroups highly or less expressed in three vertebrates (human, mouse, and zebrafish), 1 of tunicate and fly, and 1 of sponge and *C. owczarzaki* were selected.

KEGG pathway enrichment analysis

To search for genes that are highly and commonly expressed across different species, we searched for genes with TPM values of human data normalized among protein coding genes. Genes whose TPM values were >3 and >$2^{0.5}$ fold higher than mean values were defined as highly expressed genes. If a gene belonged to a differentially and highly expressed Orthogroup, the gene was regarded as shared among different species and an ancestral lineage gene. Then, we chose highly expressed ancestral genes in human and calculated how frequently enriched they were in KEGG pathways. For comparison among blood cell lineages, if a gene belonged to a Orthogroup that also contained highly expressed genes in all the species (human, mouse, and zebrafish for a vertebrate ancestor, human, mouse, zebrafish, tunicate, and sea urchin for a deuterostome ancestor, and human, mouse, zebrafish, tunicate, sea urchin, and fly for a bilaterian ancestor), the gene was regarded as shared among different species and an ancestral lineage gene. As for vertebrate blood cell lineages, Hs3, Mm1, Dr2 data were analyzed because these data containing almost all lineages, and only macrophages and neutrophils were selected in vertebrate macrophage lineage cells. As for fly, CCs were selected as mast/killer lineage cells. The selected highly expressed genes in human shared among different species were analyzed how enriched in infectious disease in KEGG (https://www.genome.jp/kegg/mapper/search.html).

TF binding estimation

We estimated TF binding sites in various organism, analyzing TF binding motif information of several animals and promoter DNA sequence data. First, TF binding motif data across different species were obtained in JASPAR database (https://jaspar.elixir.no/) and a previous report (101), and common nucleotide sequences were identified which were estimated to be conserved from ancestors. We also collected promoter DNA sequences (1000bp) upstream from transcription start sites of genes from mouse (GRCm38/Mm10), tunicate (102), sea urchin (92), fly (https://flybase.org/), and *C. owczarzaki* (103), and searched for the DNA sequences common for TFs across different species.

Chromatin accessibility analysis

To compare chromatin accessibility status across different cell lineages and maturation stages in blood cells, we analyzed public ATAC-seq data which are related to Mm2 mouse RNA-seq dataset (56) and two other data (57, 58) to cover wide lineages (*SI Appendix*, Dataset S1). ATAC peaks were normalized with peak highest at promoter region of *Cdc42* which was constitutively highly expressed across all the lineages utilizing IGV software (IGV_2.8.12).

Gene age analysis

We examined how far species shared Orthogroups containing human or mouse genes, and genes were regarded as originated from common eukaryote-prokaryote ancestors if they were shared with at least one species of the prokaryotes. If genes were not shared with any prokaryotes, but with other non-Holozoan eukaryotes, they were regarded as eukaryote genes. If genes were not originated from prokaryote or eukaryote ancestors but shared with unicellular Holozoan species, they were regarded as originated Holozoan genes. Among remained genes, those shared with sponge were regarded as Metazoan genes. Bilaterian genes, deuterostome genes, vertebrate genes, and mammalian genes were defined in the same way. Then we examined gene ages of highly and less expressed Orthogroups and genes in each cell lineages. Number of up/down-regulated Orthogroups and genes were normalized among genes in each

age, and proportions among whole Orthogroups and genes were calculated. Finally, gene age scores were calculated by comparing proportions in up- and down-regulated genes as below.

(Gene age scores) = (proportion of up-regulated genes) - (proportion of down-regulated genes)

Wright-Giemsa stain

The isolated cells were attached to microscope slides by using a Cytospin 4 (Thermo Fisher Scientific) for 5 min at 500 rpm. The slides were stained and fixed with Wright solution for 5 minutes, washed with 1/150 M phosphate buffer twice, and stained with diluted Giemsa solution for 5 minutes.

RNA-seq

Total RNA was isolated using an RNeasy kit (Qiagen) following the manufacturer's instructions. RNA-sequencing was performed by Takara Bio as follows. Libraries were prepared using SMART-Seq v4 Ultra Low Input RNA Kit for Sequencing (Takara Bio), Nextera XT DNA Library Prep Kit (Illumina), and IDT for Illumina - DNA/RNA UD Indexes, Tagmentation (Illumina). Sequencing was performed with Novaseq 6000 (Illumina), NovaSeq 6000 [S4/SP] Reagent Kit v1.5 (Illumina), NovaSeq [Xp 4-Lane/Xp 2-Lane] Kit v1.5 (Illumina), NovaSeq Control Software (version 1.7.5) (Illumina), Real Time Analysis (RTA) (version 3.4.4) (Illumina), and bcl2fastq2 (version 2.20) (Illumina).

Whole-mount in situ hybridization

Whole body *in situ* hybridization (WISH) was performed as previously described (43, 45, 104). Tunicate samples were incubated in artificial seawater with 56μg/ml of L-menthol for 10 minutes, followed by fixation with 4% of paraformaldehyde in 0.5M NaCl and 0.1 M MOPS buffer at 4℃ overnight. Then, samples were incubated in PBST with 10μg/ml proteinase K at room temperature for 25-40 minutes and washed with PBST three times. Samples were fixed again in PBSCT with 4% paraformaldehyde for 1h and washed with PBST three times. Next, samples were incubated in buffer with 50% Formamide, 5x saline-sodium citrate, 5x Denhardt's Solution, 100μg/ml yeast tRNA, and 0.1% Tween at 42℃ for 1hour, and hybridized with hybridization buffer (DIG-labeled RNA probe in the prehybridization buffer, final concentration of probe was 0.1-1μg/ml, at 42℃ for 16h, followed by a wash with PBST for 3 times. Samples were incubated in PBST with 20μg/ml of RNase at 37℃ for 30 minutes, followed by a wash with PBST twice. Then, hybridization was blocked with Blocking One solution (1ml of Blocking One and 4ml of distilled water) for 1h. Hybridized samples were labeled with anti-DIG-alkaline phosphate antibody (diluted 2000 to 3000 times with Blocking One solution, which was also diluted 20 times), and incubated with BCIP-NBT solution for 2-3 hours. Finally, RNase free water was added to stop labelling, washed with PBST, and observed with a microscope. Expression of *Tecta* (ENSCING00000010439/KY.Chr1.2359), *Dlk* (ENSCING00000021628/KY.Chr11.146), and *Foxn1/4* (ENSCING00000018677-ENSCING00000017653/KY.Chr6.670) homologs were examined.

Antibodies

The following antibodies were purchased from BioLegend: PE-FcεRIα (MAR-1; 134307), PECy7-Sca1 (D7; 108114), PECy7-ST2 (DIH9; 145315), APC-CD3ε (145-2C11; 100312), APC-CD11b (M1/70; 101212), APC-CD19 (1D3; 152410), BV421-CD11b (M1/70; 101236), BV421-CD150 (TC15-12F12.2; 115925), biotin-CD4 (RM4-5; 100508), biotin-CD8α (53-6.7; 100704), biotin-CD11b (M1/70; 101204), biotin-CD11c (N418; 117304), biotin-TER119 (TER119; 116204).

The following antibodies were purchased from eBioscience: PE-CD25 (PC61.5; 12-0251-83), PE-CD41 (eBioMWReg30; 12-0411-81), PE-TER119 (TER119; 12-5921-81), PE-F4/80 (BM8; 12-4801-80), PE-CD43 (R2/60; 12-0431-82), APC-CD135(FLT3) (A2F10; 17-1351-82), eFluor660-CD34 (RAM34; 50-0341-80), APCeFluor780-ckit (ACK2; 47-1172-82), biotin-IL7R (A7R34; 13-1271-85), biotin-NK1.1 (PK136; 13-5941-85).

The following antibodies were purchased from BD Biosciences: FITC-streptavidin (554060), PE-FcR (2.4G2; 553145), PECy7-B220 (RA3-6B2; 561881), APC-ckit (2B8; 553356), APC-Gr1 (RB6-8C5; 553129), APCCy7-streptavidin (554063), V450-strepavidin (560797), biotin-CD3ε (145-2C11; 553060), biotin-CD19 (1D3; 553784), biotin-FcR (2.4G2; 553143).

The following antibodies were purchased from TONBO Biosciences: PECy7-CD11c (N418; 60-0114-U100).

Growth factors
Recombinant murine SCF, Flt3-Ligand (L), IL-3, IL-7, IL-33, TPO, and human EPO were purchased from Peprotech.
Isolation of progenitors
Single-cell suspensions of the thymus or femoral and tibial BM from wild-type mice were prepared. DN1-3 cells were isolated from thymocytes and GMPs, MEPs, proB cells, and prepro-B cells were isolated from BM cells. The gating strategy is shown in *SI Appendix*, Fig. S8.
In vitro culture of progenitors
The isolated progenitors were co-cultured with TSt4-DLL1 cells at 37℃ and 5% CO2 for 14 days in RPMI1640 medium supplemented with 10% FBS, 2 mM L-glutamine, 1 mM sodium pyruvate, 2 mg/mL sodium bicarbonate, 0.1 mM nonessential amino acid solution, 5 × 10−5 M 2-ME, 100 mg/mL streptomycin, 100 U/mL penicillin, 20 ng/mL of SCF, Flt3-L 50ng/ml of IL-3, 10ng/ml of IL-7 and IL-33. For MEPs, 2 U/mL of EPO and 25 ng/mL of TPO were added. The numbers of cultured progenitors were 100 cells (GMP, LMPP, DN1, and DN2) or 1000 cells (MEP, DN3, preproB, and proB) per well.
Enforced gene expression by knock-in transformation in *C. owczarzaki*
Knock-in transformation by homologous recombination was performed referring a previous report (105), with one modification of length of homology arms from 90 bp to 1000 bp. In brief, 6 x 10^5 cells/well were seeded on a 24-well plate one day before transformation. Culture medium was replaced with 800 μl of transfection medium of Scheider's Drosophila Medium (ThermoFisher scientific 21720024) supplemented with 10% FBS and 25 μg/mL ampicillin. After 10 minutes incubation at room temperature, medium was replaced with another 500 μl of transfection medium. Transfection mix was prepared mixing 1μg linearized gene, 3 μL of TransIT-X2, and 100μl of Opti-MEM. After 5 minutes incubation at room temperature, 70μl of transfection mix was added to the medium. One day after transfection (day 1), transfected cells were selected by adding 50 μg/ml of hygromycin. Hygromycin concentration was increased to 100 μg/ml at day 4, and knocked-in cells were selected. For evaluating cell aggregation, control and *Fos* (CAOG_02712) knocked-in 1 x 10^4 cells were seeded in $25cm^2$ flask with 10ml medium, and observed 7-9 days later with Zeis AXIO observer Z1 microscope. Numbers of aggregations were manually counted. For evaluating cell proliferation rate, control and *Myb* (CAOG_00301) knocked-in 1x10^4 cells were seeded in each well of 48-well plate with 400μl medium, and cell numbers were counted 4 days later. For cell numbers at day 7, cells were transferred to new wells with 1/100 cell concentration at the fourth day of culture, and cell numbers were counted 3 days later.
Statistical analysis
Continuous variables were compared using two-tailed t-tests, respectively. Statistical analyses for correlation of gene expression levels were performed using Anaconda 24.1.2, Jupyter Notebook 7.0.8., numpy 1.26.4, and pandas 2.1.4.

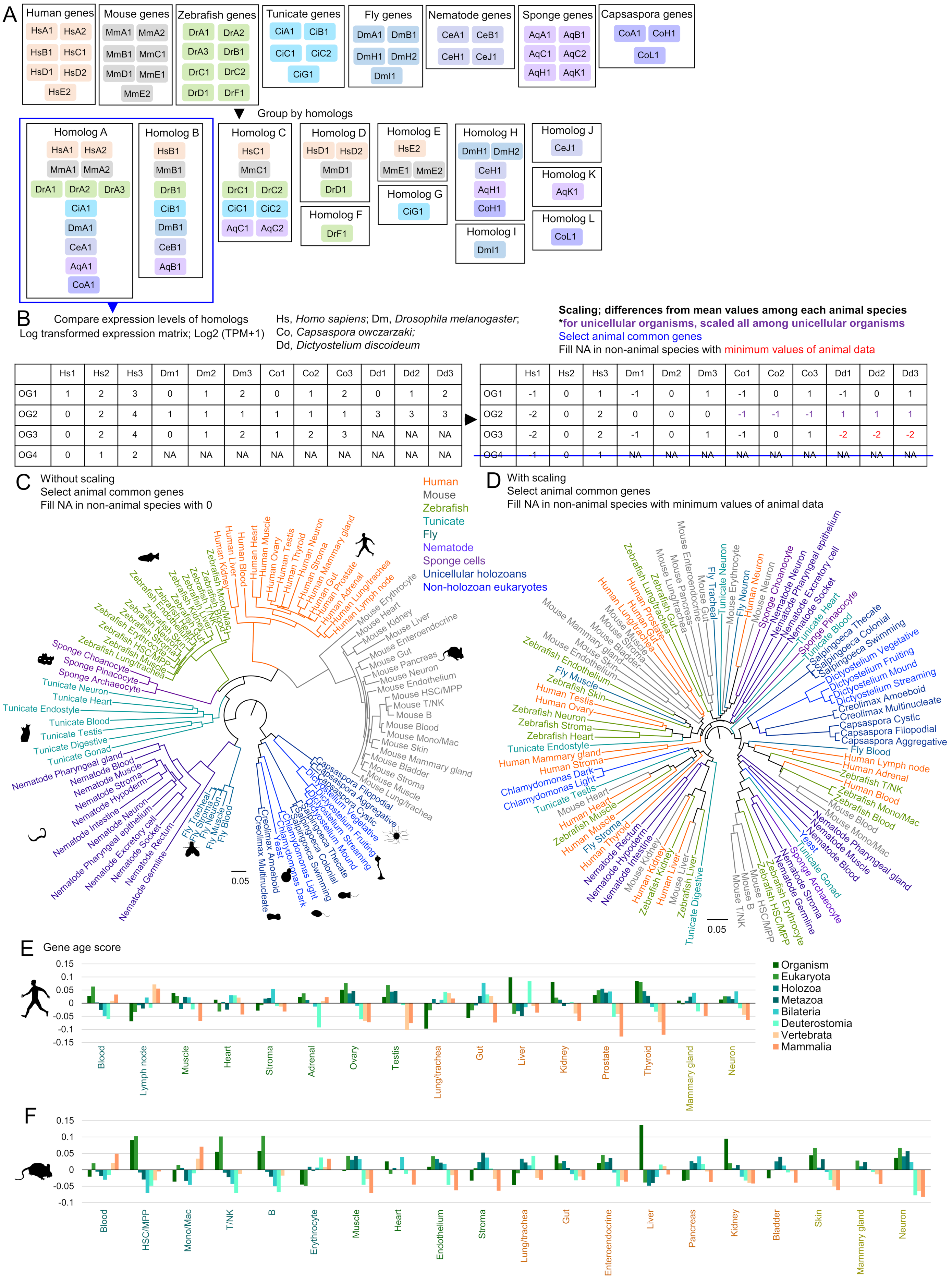

A
Human genes
Mouse genes
Zebrafish genes
Tunicate genes
Fly genes
Nematode genes
Sponge genes
Capsaspora genes
Group by homologs
Homolog A
Homolog B
Homolog C
Homolog D
Homolog E
Homolog F
Homolog G
Homolog H
Homolog I
Homolog J
Homolog K
Homolog L
B
Compare expression levels of homologs
Log transformed expression matrix; Log2 (TPM+1)
Hs, Homo sapiens; Dm, Drosophila melanogaster; Co, Capsaspora owczarzaki; Dd, Dictyostelium discoideum
Scaling; differences from mean values among each animal species
*for unicellular organisms, scaled all among unicellular organisms
Select animal common genes
Fill NA in non-animal species with minimum values of animal data
	Hs1	Hs2	Hs3	Dm1	Dm2	Dm3	Co1	Co2	Co3	Dd1	Dd2	Dd3
OG1	1	2	3	0	1	2	0	1	2	0	1	2
OG2	0	2	4	1	1	1	1	1	1	3	3	3
OG3	0	2	4	0	1	2	1	2	3	NA	NA	NA
OG4	0	1	2	NA	NA	NA	NA	NA	NA	NA	NA	NA
	Hs1	Hs2	Hs3	Dm1	Dm2	Dm3	Co1	Co2	Co3	Dd1	Dd2	Dd3
OG1	-1	0	1	-1	0	1	-1	0	1	-1	0	1
OG2	-2	0	2	0	0	0	-1	-1	-1	1	1	1
OG3	-2	0	2	-1	0	1	-1	0	1	-2	-2	-2
OG4	-1	0	1	NA	NA	NA	NA	NA	NA	NA	NA	NA
C
Without scaling
Select animal common genes
Fill NA in non-animal species with 0
Human
Mouse
Zebrafish
Tunicate
Fly
Nematode
Sponge cells
Unicellular holozoans
Non-holozoan eukaryotes
0.05
D
With scaling
Select animal common genes
Fill NA in non-animal species with minimum values of animal data
0.05
E
Gene age score
Organism
Eukaryota
Holozoa
Metazoa
Bilateria
Deuterostomia
Vertebrata
Mammalia
Blood
Lymph node
Muscle
Heart
Stroma
Adrenal
Ovary
Testis
Lung/trachea
Gut
Liver
Kidney
Prostate
Thyroid
Mammary gland
Neuron
F
Blood
HSC/MPP
Mono/Mac
T/NK
B
Erythrocyte
Muscle
Heart
Endothelium
Stroma
Lung/trachea
Gut
Enteroendocrine
Liver
Pancreas
Kidney
Bladder
Skin
Mammary gland
Neuron

Fig. S1. Cross species comparison of transcriptome data. (***A***) Method to compare transcriptome data among different species. All protein-coding genes of were classified into homolog groups with OrthoFinder (79), and expression levels of homologs conserved among the analyzed species were compared. (***B***) A simplified example to integrate and compare transcriptome data of different datasets and species. (***C***-***D***) Phylogenetic trees colored with species with (C) or without (D) scaling values. Scaling values, reduced inter-species/dataset differences and enable to compare cell lineages. (***E***-***F***) Gene age score analysis for human (E) and mouse (F) data showed that blood cells expressed old genes inherited from unicellular ancestors. Mac, macrophage; Mono, monocyte; Neut, neutrophil.

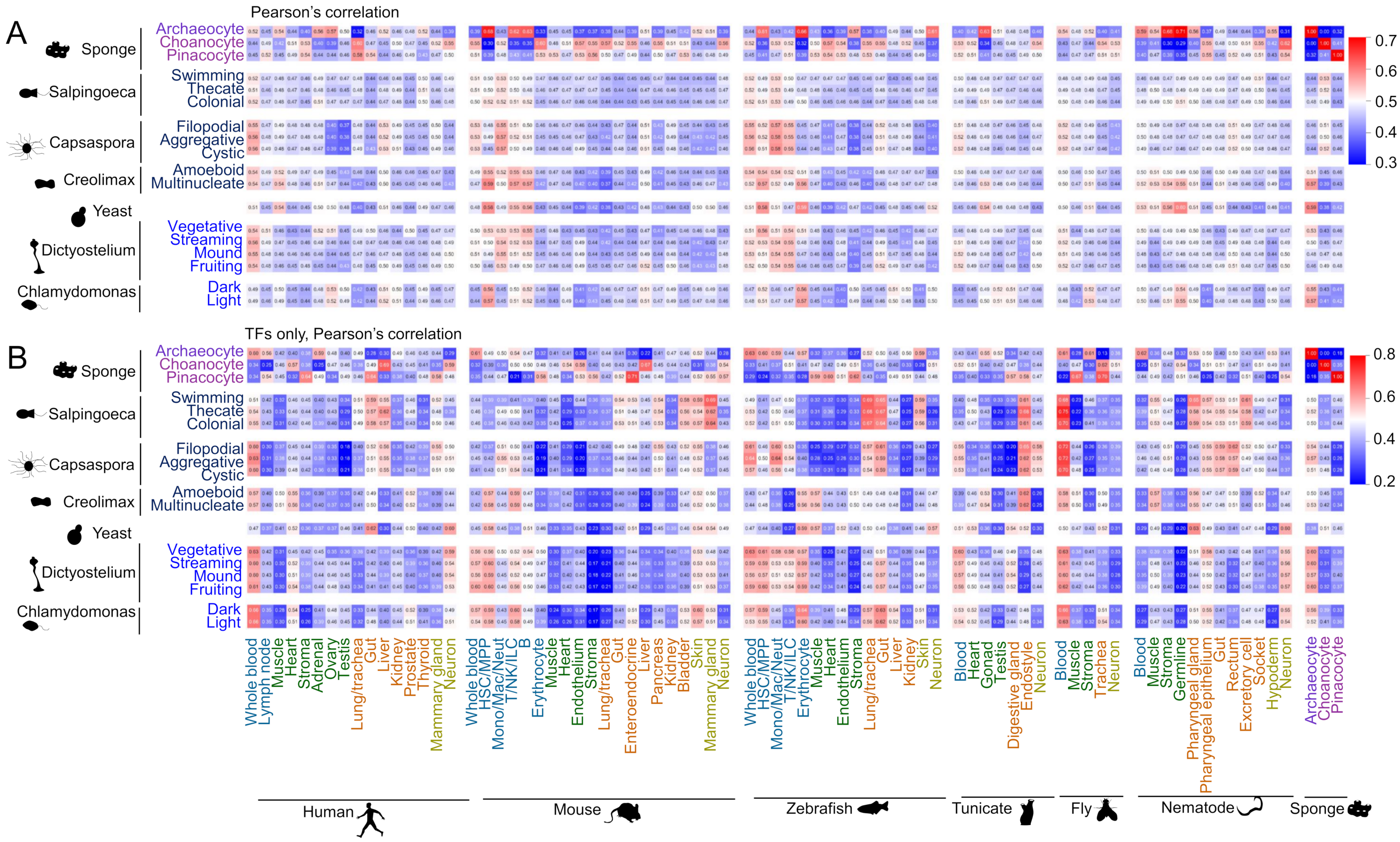

A
Pearson's correlation
B
TFs only, Pearson's correlation
Sponge
Archaeocyte
Choanocyte
Pinacocyte
Salpingoeca
Swimming
Thecate
Colonial
Capsaspora
Filopodial
Aggregative
Cystic
Creolimax
Amoeboid
Multinucleate
Yeast
Dictyostelium
Vegetative
Streaming
Mound
Fruiting
Chlamydomonas
Dark
Light
0.7
0.6
0.5
0.4
0.3
0.8
0.2
Whole blood
Lymph node
Muscle
Heart
Stroma
Adrenal
Ovary
Testis
Lung/trachea
Gut
Liver
Kidney
Prostate
Thyroid
Mammary gland
Neuron
HSC/MPP
Mono/Mac/Neut
T/NK/ILC
B
Erythrocyte
Endothelium
Enteroendocrine
Pancreas
Bladder
Skin
Blood
Gonad
Digestive gland
Endostyle
Trachea
Germline
Pharyngeal gland
Pharyngeal epithelium
Rectum
Excretory cell
Socket
Hypoderm
Human
Mouse
Zebrafish
Tunicate
Fly
Nematode
Sponge

Fig. S2. The initial blood cells were similar to unicellular organisms. (***A-B***) Correlation values for cells lineages between metazoan species and unicellular organisms based on 1610 Orthogroups (A) or 25 TFs (B). Mac, macrophage; Mono, monocyte; Neut, neutrophil.

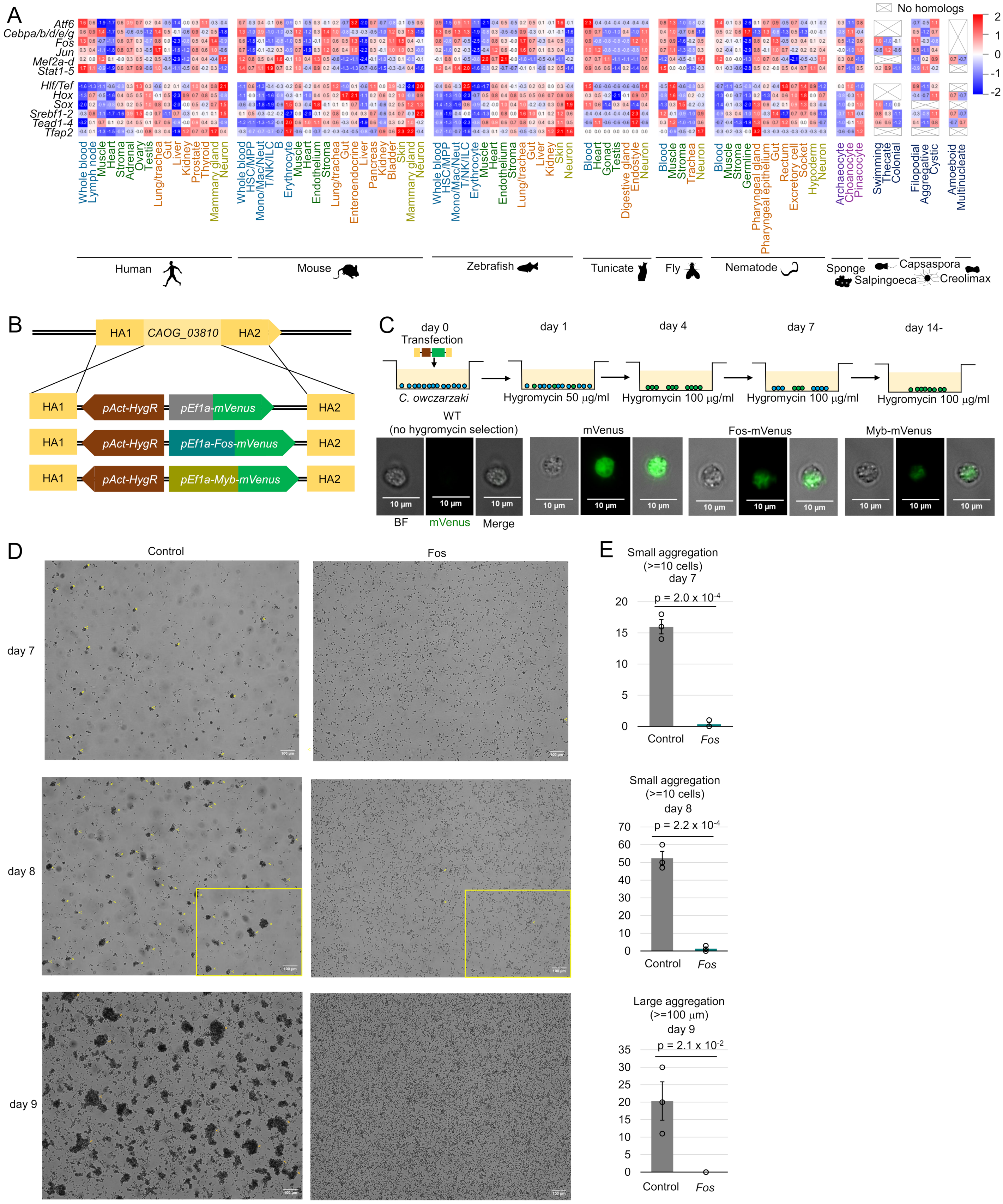
A
Atf6
Cebpa/b/d/e/g
Fos
Jun
Mef2a-d
Stat1-5
Hlf/Tef
Hox
Sox
Srebf1-2
Tead1-4
Tfap2
No homologs
2
1
0
-1
-2
Whole blood
Lymph node
Muscle
Heart
Stroma
Adrenal
Ovary
Testis
Lung/trachea
Gut
Liver
Kidney
Prostate
Thyroid
Mammary gland
Neuron
HSC/MPP
Mono/Mac/Neut
T/NK/ILC
B
Erythrocyte
Endothelium
Enteroendocrine
Pancreas
Bladder
Skin
Blood
Gonad
Digestive gland
Endostyle
Trachea
Germline
Pharyngeal gland
Pharyngeal epithelium
Rectum
Excretory cell
Socket
Hypoderm
Archaeocyte
Choanocyte
Pinacocyte
Swimming
Thecate
Colonial
Filopodial
Aggregative
Cystic
Amoeboid
Multinucleate
Human
Mouse
Zebrafish
Tunicate
Fly
Nematode
Sponge
Salpingoeca
Capsaspora
Creolimax
B
HA1
CAOG_03810
HA2
pAct-HygR
pEf1a-mVenus
pEf1a-Fos-mVenus
pEf1a-Myb-mVenus
C
day 0
Transfection
day 1
day 4
day 7
day 14-
C. owczarzaki
Hygromycin 50 μg/ml
Hygromycin 100 μg/ml
WT
(no hygromycin selection)
mVenus
Fos-mVenus
Myb-mVenus
10 μm
BF
mVenus
Merge
D
Control
Fos
day 7
day 8
day 9
E
Small aggregation
(>=10 cells)
day 7
p = 2.0 x 10-4
Control
Fos
Small aggregation
(>=10 cells)
day 8
p = 2.2 x 10-4
Large aggregation
(>=100 μm)
day 9
p = 2.1 x 10-2

Fig. S3. TFs driving blood-cell or amoeboid-stage program among holozoan species. (***A***) Heat map of Z-scores of expression levels [log2(TPM+1)] of TFs commonly expressed or repressed in vertebrate, tunicate, fly, nematode, and sponge blood cells. TFs commonly expressed bilaterian blood cells and filopodial stage of *C. owczarzaki* are also shown. Z-scores were calculated within each species, respectively. (***B***) Schematic illustration of knock-in transformation with homologous recombination in *C. owczarzaki*. Hygromycin resistant gene and *mVenus* (control), *Fos-mVenus, or Myb-mVenus* gene were knocked into *CAOG_03810* locus with 1000 bp homology arms. (***C***) *C. owczarzaki* cells were transfected with linearized nucleotides, and transfected cells were selected by adding hygromycin. Transformed cells which expressing knock-in genes over 7 days were further selected by continuous hygromycin selection. Selection and expression of knock-in genes were verified by mVenus expression. (***D***) Representative images of control and *Fos* expressing *C. owczarzaki* cells after 7-9 days culture (n = 3). Aggregations with 10 or more cells and those larger than 100 μm were indicated with yellow and orange marks, respectively. The yellow boxed areas of images are enlarged in Fig. 1D. (***E***) Numbers of aggregations shown with mean values ± SE of biological replicates (n = 3). HA, homology arm; HygR, hygromycin resistant gene; Mac, macrophage; Mono, monocyte; Neut, neutrophil.

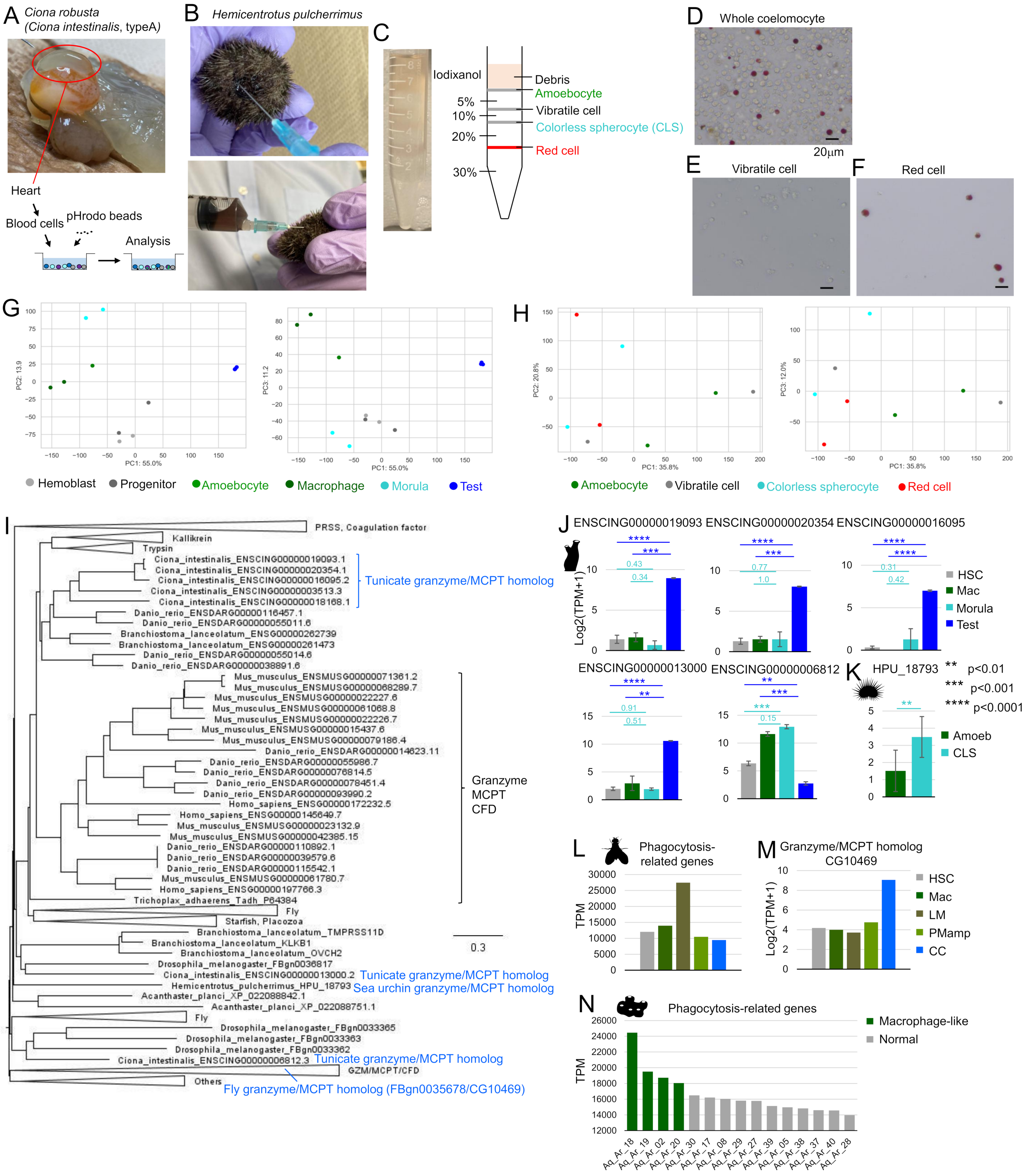
A Ciona robusta (Ciona intestinalis, typeA)
Heart
Blood cells
pHrodo beads
Analysis
B Hemicentrotus pulcherrimus
C
Iodixanol
5%
10%
20%
30%
Debris
Amoebocyte
Vibratile cell
Colorless spherocyte (CLS)
Red cell
D Whole coelomocyte
20μm
E Vibratile cell
F Red cell
G
PC1: 55.0%
PC2: 13.9
PC3: 11.2
Hemoblast
Progenitor
Amoebocyte
Macrophage
Morula
Test
H
PC1: 35.8%
PC2: 20.8%
PC3: 12.0%
Amoebocyte
Vibratile cell
Colorless spherocyte
Red cell
I
PRSS, Coagulation factor
Kallikrein
Trypsin
Ciona_intestinalis_ENSCING00000019093.1
Ciona_intestinalis_ENSCING00000020354.1
Ciona_intestinalis_ENSCING00000016095.2
Ciona_intestinalis_ENSCING00000003513.3
Ciona_intestinalis_ENSCING00000018168.1
Tunicate granzyme/MCPT homolog
Danio_rerio_ENSDARG00000116457.1
Danio_rerio_ENSDARG00000055011.6
Branchiostoma_lanceolatum_ENSG00000262739
Branchiostoma_lanceolatum_ENSG00000261473
Danio_rerio_ENSDARG00000055014.6
Danio_rerio_ENSDARG00000038891.6
Mus_musculus_ENSMUSG00000071361.2
Mus_musculus_ENSMUSG00000068289.7
Mus_musculus_ENSMUSG00000022227.6
Mus_musculus_ENSMUSG00000061068.8
Mus_musculus_ENSMUSG00000022226.7
Mus_musculus_ENSMUSG00000015437.6
Mus_musculus_ENSMUSG00000079186.4
Danio_rerio_ENSDARG00000014623.11
Danio_rerio_ENSDARG00000055986.7
Danio_rerio_ENSDARG00000076814.5
Danio_rerio_ENSDARG00000078451.4
Danio_rerio_ENSDARG00000093990.2
Homo_sapiens_ENSG00000172232.5
Homo_sapiens_ENSG00000145649.7
Mus_musculus_ENSMUSG00000023132.9
Mus_musculus_ENSMUSG00000042385.15
Danio_rerio_ENSDARG00000110892.1
Danio_rerio_ENSDARG00000039579.6
Danio_rerio_ENSDARG00000115542.1
Mus_musculus_ENSMUSG00000061780.7
Homo_sapiens_ENSG00000197766.3
Trichoplax_adhaerens_Tadh_P64384
Granzyme
MCPT
CFD
Fly
Starfish, Placozoa
Branchiostoma_lanceolatum_TMPRSS11D
Branchiostoma_lanceolatum_KLKB1
Branchiostoma_lanceolatum_OVCH2
Drosophila_melanogaster_FBgn0036817
Ciona_intestinalis_ENSCING00000013000.2 Tunicate granzyme/MCPT homolog
Hemicentrotus_pulcherrimus_HPU_18793 Sea urchin granzyme/MCPT homolog
Acanthaster_planci_XP_022088842.1
Acanthaster_planci_XP_022088751.1
0.3
Fly
Drosophila_melanogaster_FBgn0033365
Drosophila_melanogaster_FBgn0033363
Drosophila_melanogaster_FBgn0033362
Ciona_intestinalis_ENSCING00000006812.3 Tunicate granzyme/MCPT homolog
GZM/MCPT/CFD
Others
Fly granzyme/MCPT homolog (FBgn0035678/CG10469)
J ENSCING00000019093 ENSCING00000020354 ENSCING00000016095
Log2(TPM+1)
HSC
Mac
Morula
Test
ENSCING00000013000 ENSCING00000006812
K HPU_18793
** p<0.01
*** p<0.001
**** p<0.0001
Amoeb
CLS
L Phagocytosis-related genes
TPM
M Granzyme/MCPT homolog CG10469
Log2(TPM+1)
HSC
Mac
LM
PMamp
CC
N Phagocytosis-related genes
TPM
Macrophage-like
Normal
Aq_Ar_18
Aq_Ar_19
Aq_Ar_02
Aq_Ar_20
Aq_Ar_30
Aq_Ar_17
Aq_Ar_08
Aq_Ar_29
Aq_Ar_27
Aq_Ar_39
Aq_Ar_05
Aq_Ar_38
Aq_Ar_37
Aq_Ar_40
Aq_Ar_28

Fig. S4. Transcriptomic analysis of tunicates and sea urchins blood cells. (***A***) Tunicate blood cells were aspirated by cardiac puncture (8). Collected blood cells were incubated with pHrodo beads and analyzed by flow cytometry. (***B***-***C***) Sea urchin blood cells (coelomocytes) were aspirated by coelomic-cavity puncture (B). Collected blood cells were separated by density gradient centrifugation with iodixanol into the indicated fractions (C). (***D***-***F***) Representative images of whole coelomocytes (D), vibratile cells (E), and red cells (F). (***G***-***H***) Principal component (PC) analysis of tunicate (G) and sea urchin (H) blood cells with PC1+PC2 and PC1+PC3. (***I***) Phylogenetic tree of homologs containing granzymes and mast cell proteases (OG0000005) generated by the OrthoFinder algorithm. Kallikrein and trypsin homologs and branches exclusively containing zebrafish, starfish, fly, and Placozoa homologs were collapsed. (***J***-***K***) Expression levels of granzyme and MCPT homologs in tunicate (J) and sea urchin (K) blood cells. As for tunicate homologs, five genes with significant differences were shown. ENSCING00000020354, ENSCING00000019093, and ENSCING00000016095 were regarded as transcript variants in the KY genome model. (***L***-***M***) Expression levels of phagocytosis-related genes (L) and the granzyme/mast-cell-protease homolog CG10469 [M, log2(TPM+1)] in fly blood cells. (***N***) Expression levels of phagocytosis-related genes in sponge archaeocytes. Four archaeocytes highly expressing phagocytosis related genes >18000 were regarded as macrophage-like archaeocytes. Amoeb, amoebocyte; Mac, macrophage.

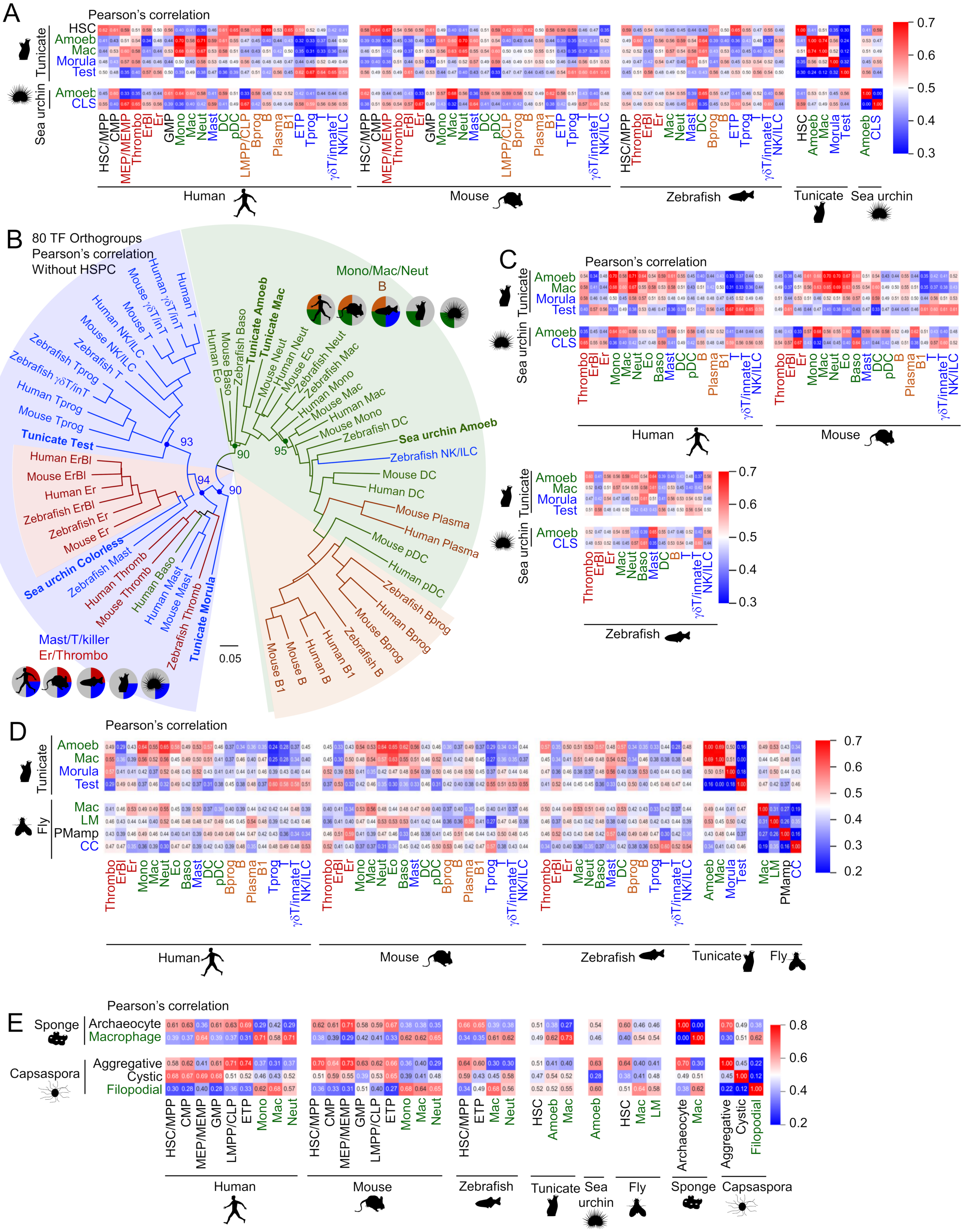
A
Pearson's correlation
HSC
Amoeb
Mac
Morula
Test
Tunicate
Amoeb
CLS
Sea urchin
HSC/MPP
CMP
MEP/MEMP
Thrombo
ErBl
Er
GMP
Mono
Mac
Neut
Mast
DC
pDC
LMPP/CLP
Bprog
B
Plasma
B1
ETP
Tprog
T
γδT/innateT
NK/ILC
Human
Mouse
Zebrafish
Tunicate
Sea urchin
0.7
0.6
0.5
0.4
0.3
B
80 TF Orthogroups
Pearson's correlation
Without HSPC
Mono/Mac/Neut
B
Mast/T/killer
Er/Thrombo
0.05
93
90
95
94
90
Human T
Human γδT/inT
Mouse γδT/inT
Mouse T
Human NK/ILC
Mouse NK/ILC
Zebrafish T
Zebrafish Tprog
Zebrafish γδT/inT
Human Tprog
Mouse Tprog
Tunicate Test
Human ErBl
Mouse ErBl
Human Er
Zebrafish ErBl
Zebrafish Er
Mouse Er
Sea urchin Colorless
Zebrafish Mast
Human Thromb
Mouse Thromb
Human Baso
Human Mast
Mouse Mast
Zebrafish Thromb
Tunicate Morula
Human Eo
Mouse Baso
Zebrafish Baso
Tunicate Amoeb
Tunicate Mac
Mouse Neut
Human Neut
Mouse Eo
Zebrafish Neut
Zebrafish Mac
Zebrafish Mono
Human Mono
Mouse Mac
Human Mac
Mouse Mono
Zebrafish DC
Sea urchin Amoeb
Zebrafish NK/ILC
Mouse DC
Human DC
Mouse Plasma
Human Plasma
Mouse pDC
Human pDC
Zebrafish Bprog
Human Bprog
Mouse Bprog
Zebrafish B
Human B1
Human B
Mouse B
Mouse B1
C
Pearson's correlation
Amoeb
Mac
Morula
Test
Tunicate
Amoeb
CLS
Sea urchin
Thrombo
ErBl
Er
Mono
Mac
Neut
Eo
Baso
Mast
DC
pDC
B
Plasma
B1
T
γδT/innateT
NK/ILC
Human
Mouse
Zebrafish
0.7
0.6
0.5
0.4
0.3
D
Pearson's correlation
Amoeb
Mac
Morula
Test
Tunicate
Mac
LM
PMamp
CC
Fly
Thrombo
ErBl
Er
Mono
Mac
Neut
Eo
Baso
Mast
DC
pDC
Bprog
B
Plasma
B1
Tprog
T
γδT/innateT
NK/ILC
Human
Mouse
Zebrafish
Tunicate
Fly
0.7
0.6
0.5
0.4
0.3
0.2
E
Pearson's correlation
Sponge
Archaeocyte
Macrophage
Capsaspora
Aggregative
Cystic
Filopodial
HSC/MPP
CMP
MEP/MEMP
GMP
LMPP/CLP
ETP
Mono
Mac
Neut
HSC
Amoeb
LM
Archaeocyte
Human
Mouse
Zebrafish
Tunicate
Sea urchin
Fly
Sponge
Capsaspora
0.8
0.6
0.4
0.2

Fig. S5. Estimated phylogeny of blood cell lineages. (***A***, ***C***-***E***) Correlation values between blood cell lineages among deuterostome (A, C) bilaterian (D) and holozoan species with (A, D, E) or without (C) HSCs/progenitors. (***B***) Neighbour-joining phylogenetic trees of blood cell lineages based on TFs among deuterostome species without HSCs/progenitors. Nodal support numbers of adjusted unbiassed bootstrap values at key bifurcations are shown with colored numbers and circles. Cell lineages included in each clade are shown with colored quarter circles. The macrophage, mast/killer-cell, B-cell, and erythrocyte/thrombocyte clades are highlighted with green, blue, orange, and red background, respectively. Amoeb, amoebocyte; Baso, basophil; Bprog, B-cell progenitor; CLP, common lymphoid progenitor; Eo, eosinophil; Er, erythrocyte; GMP, granulocyte-monocyte progenitor; Mac, macrophage; Mono, monocyte; MPP, multipotent progenitor; Neut, neutrophil; PMamp, plasmatocyte expressing antimicrobial peptides; Thromb, thrombocyte; Tprog, T-cell progenitor.

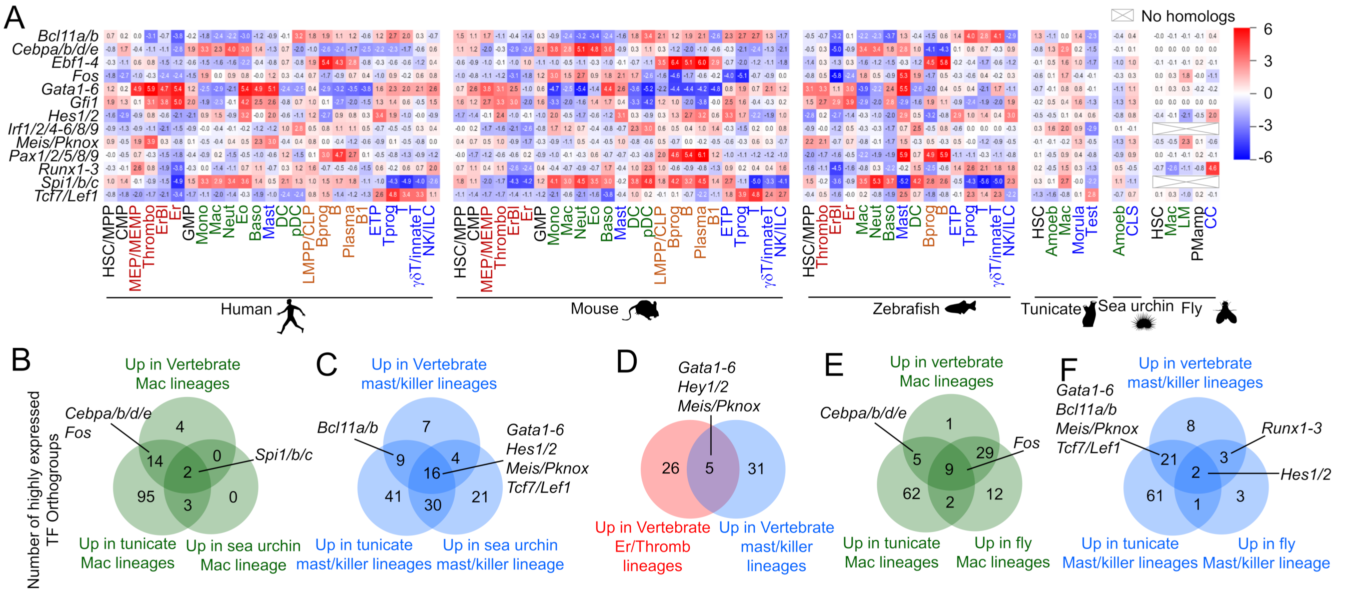

G

Gene	Human	Mouse	Zebrafish	Tunicate	Sea urchin	Fly	Sponge	Capsaspora
Bcl11a/b	2	2	4	1	2	1	0	0
Cebpa/b/d/e	4	4	4	1	1	1	1	1
Ebf1-4	4	4	7	1	1	1	1	1
Gata1-6	8	6	9	2	2	5	1	1
Fos	6	6	11	3	2	2	1	2
Hes1/2	3	2	9	3	10	2	0	0
Ikzf1-4	4	4	3	1	0	0	0	0
Irf1/2/4-6/8/9	7	7	12	2	3	0	2	0
Meis1-3, Pknox1/2	5	5	9	2	2	2	1	2
Pax1/2/5/8/9	5	5	7	1	3	3	1	0
Rora/b/g	3	3	9	1	0	1	0	0
Runx1-3	3	3	5	1	3	4	1	1
Spi1/B/C	4	3	4	1	1	0	0	0
Tcf7/7l1/7l2, Lef1	4	4	6	1	1	1	1	0
Perforin	1	1	5	0	0	0	0	0

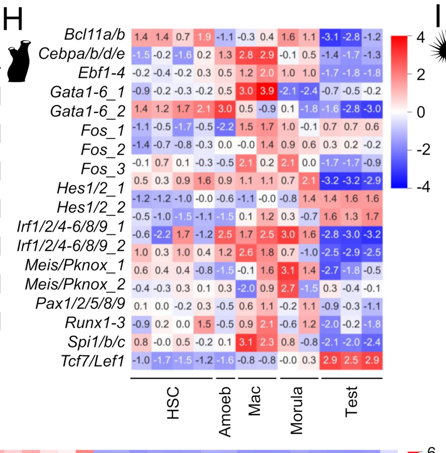

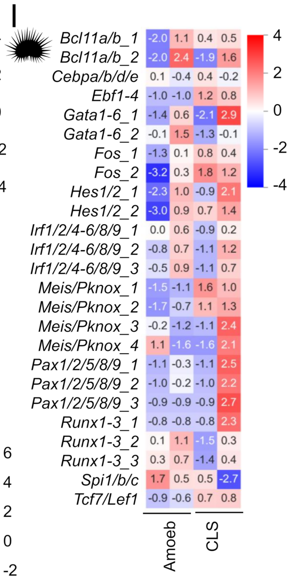

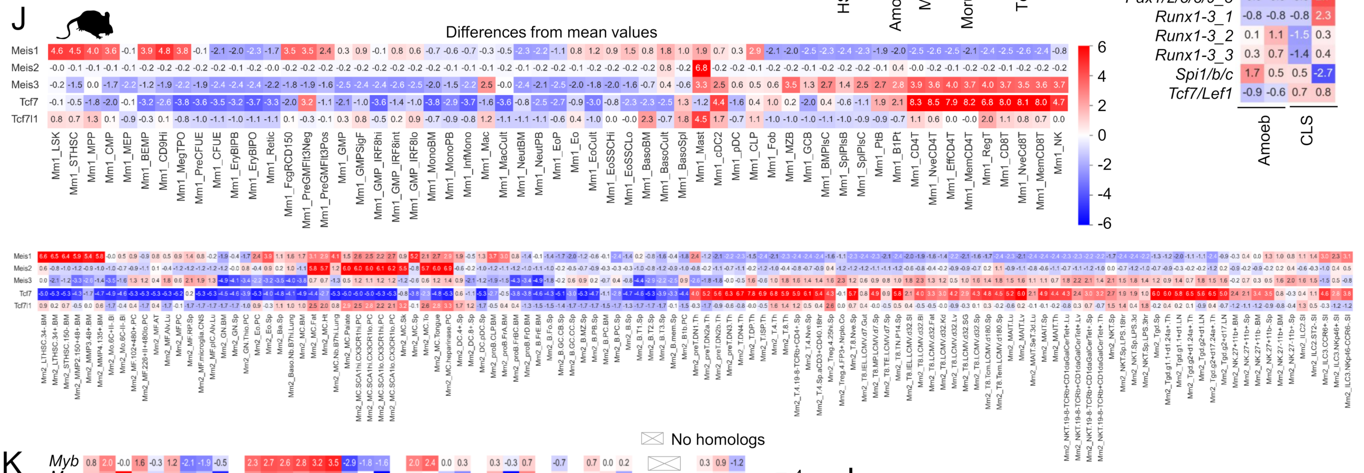

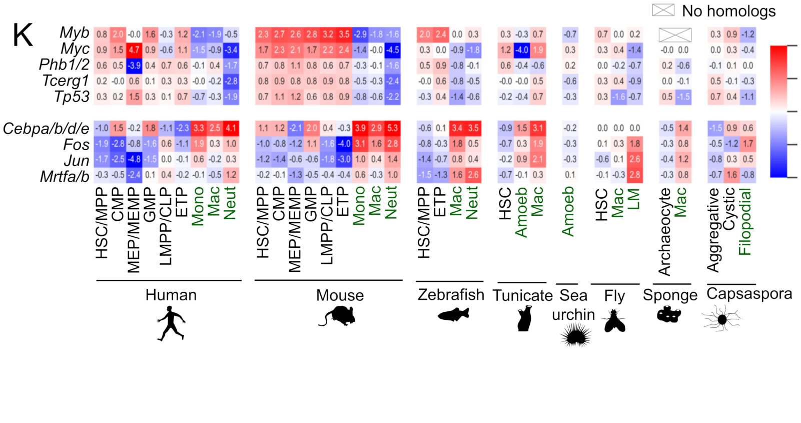

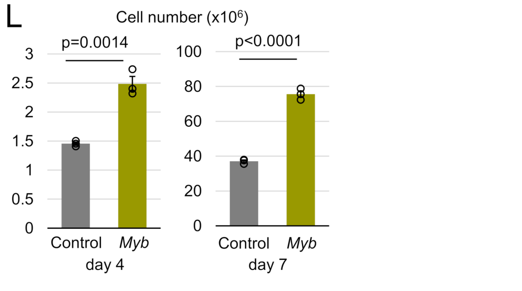

Fig. S6. Detailed expression levels of TFs of blood cell lineages. (***A***) Heat map of expression levels [log2(TPM+1), differences from mean values] of TFs in vertebrate, tunicate, sea urchin, and fly blood cells. TPM were normalized among Orthogroups shared with human, mouse, zebrafish, tunicate, and sea urchin. (***B***-***F***) Venn diagrams of numbers of highly expressed TF Orthogroups (>$2^{0.5}$). Numbers of Orthogroups highly expressed in deuterostome macrophage lineage cells (B), deuterostome killer/mast lineage cells (C), erythrocyte/thrombocyte lineage cells or killer/mast lineage cells (D), bilaterian macrophage lineage cells (E), bilaterian killer/mast lineage cells (F) are shown. (***G***) The number of homologs of TFs common in hematopoiesis and known to be important in blood cell lineages. The numbers of perforin homologs are also shown. (***H***-***I***) Heat map of expression levels [log2(TPM+1), differences from mean values] of TFs in tunicate (H) and sea urchin (I) blood cells. TPM values with multiple homologs were examined separately. n=2-3 with pooled blood cells from several dozen tunicates for blood cells and 3-4 tunicates for test cells. n=2 with pooled blood cells from two sea urchin coelomocytes. (***J***) Heat map of expression levels [log2(TPM+1)] of TFs in a mouse Mm1 dataset covering a wider range of blood cells (84) and an Mm2 dataset covering HSC/MPPs, differentiated immune cells, and their progenitors (56). Differences from mean values are shown. (***K***) Heat map of expression levels [log2(TPM+1), differences from mean values] of TFs in vertebrate, tunicate, fly, and sponge blood cells and *C. owczarzaki*. TPM were normalized among Orthogroups shared with human, mouse, zebrafish, tunicate, sea urchin, fly and *C. owczarzaki*. (***L***) Cell numbers of control and *Myb* knocked-in *C. owczarzaki* cultured for 4 and 7 days starting from 1×10^4 cells. Mean values ± SE of replicates (n = 3). Amoeb, amoebocyte; Baso, basophil; Bprog, B-cell progenitor; CLP, common lymphoid progenitor; Eo, eosinophil; Er, erythrocyte; GMP, granulocyte-monocyte progenitor; Mac, macrophage; Mono, monocyte; MPP, multipotent progenitor; Neut, neutrophil; PMamp, plasmatocyte expressing antimicrobial peptides; Thromb, thrombocyte; Tprog, T-cell progenitor.

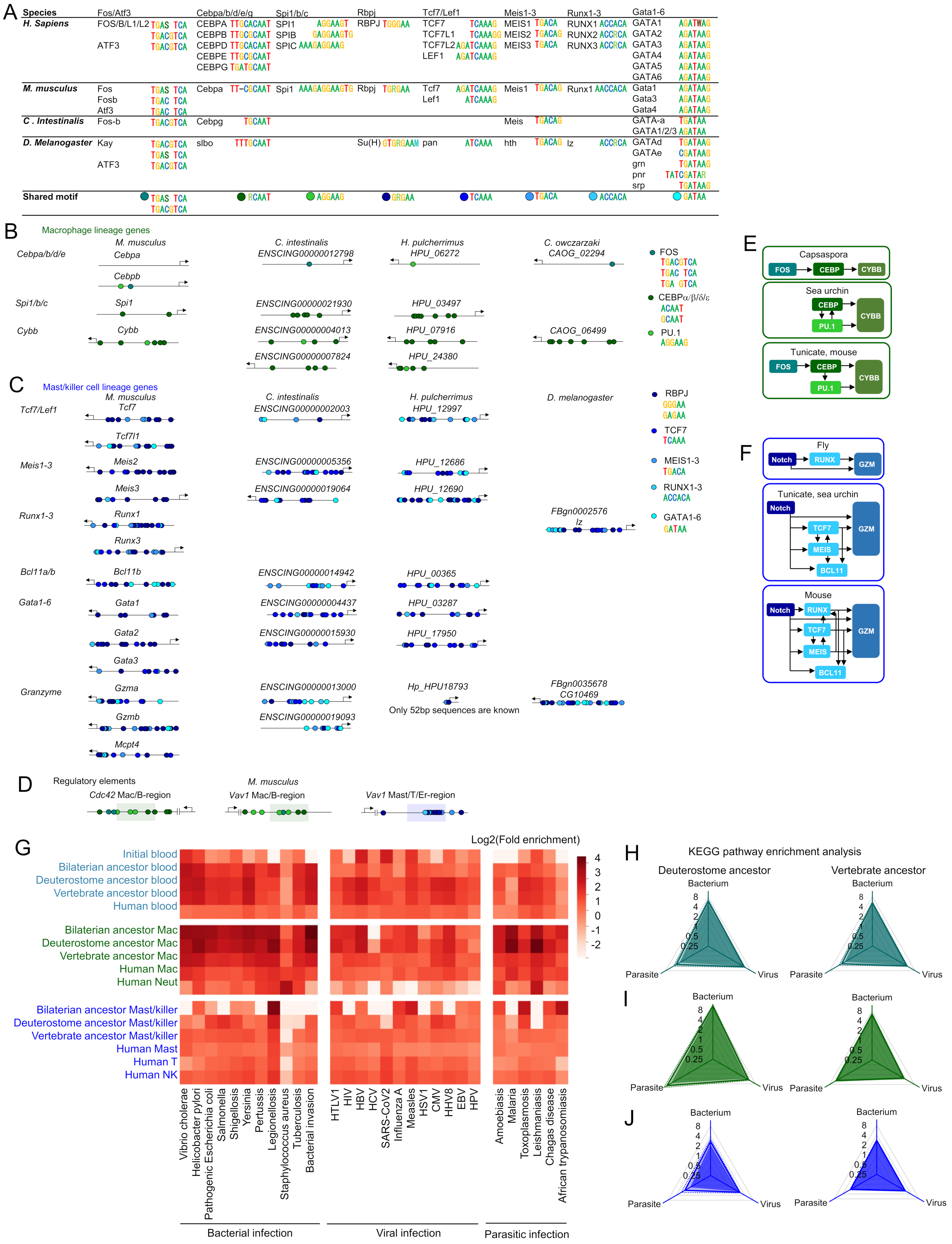
A
Species
Fos/Atf3
Cebpa/b/d/e/g
Spi1/b/c
Rbpj
Tcf7/Lef1
Meis1-3
Runx1-3
Gata1-6
H. Sapiens
M. musculus
C . Intestinalis
D. Melanogaster
Shared motif
B
Macrophage lineage genes
M. musculus
C. intestinalis
H. pulcherrimus
C. owczarzaki
Cebpa/b/d/e
Spi1/b/c
Cybb
FOS
CEBPα/β/δ/ε
PU.1
C
Mast/killer cell lineage genes
D. melanogaster
Tcf7/Lef1
Meis1-3
Runx1-3
Bcl11a/b
Gata1-6
Granzyme
Only 52bp sequences are known
RBPJ
TCF7
MEIS1-3
RUNX1-3
GATA1-6
D
Regulatory elements
Cdc42 Mac/B-region
Vav1 Mac/B-region
Vav1 Mast/T/Er-region
E
Capsaspora
Sea urchin
Tunicate, mouse
F
Fly
Tunicate, sea urchin
Mouse
G
Log2(Fold enrichment)
Initial blood
Bilaterian ancestor blood
Deuterostome ancestor blood
Vertebrate ancestor blood
Human blood
Bilaterian ancestor Mac
Deuterostome ancestor Mac
Vertebrate ancestor Mac
Human Mac
Human Neut
Bilaterian ancestor Mast/killer
Deuterostome ancestor Mast/killer
Vertebrate ancestor Mast/killer
Human Mast
Human T
Human NK
Vibrio cholerae
Helicobacter pylori
Pathogenic Escherichia coli
Salmonella
Shigellosis
Yersinia
Pertussis
Legionellosis
Staphylococcus aureus
Tuberculosis
Bacterial invasion
HTLV1
HIV
HBV
HCV
SARS-CoV2
Influenza A
Measles
HSV1
CMV
HHV8
EBV
HPV
Amoebiasis
Malaria
Toxoplasmosis
Leishmaniasis
Chagas disease
African trypanosomiasis
Bacterial infection
Viral infection
Parasitic infection
H
KEGG pathway enrichment analysis
Deuterostome ancestor
Vertebrate ancestor
Bacterium
Parasite
Virus
I
J

Fig. S7. Estimation of TF binding sites and anti-pathogen function of ancestral blood cells. (***A***) TF binding motives in human, mouse, tunicate and fly. Common nucleotide sequences are shown as estimated ancestral binding motives. (***B-D***) Candidates for TF binding sites at promoter regions 1000bp upstream from transcription start sites of macrophage (B) and mast/killer (C) lineage genes, and regulatory elements of *Cdc42* and *Vav1* (D). Each colored circles shows detected nucleotide sequences of TF binding motives shared among human, mouse, tunicate, and fly. Regions with ATAC-peaks in Fig. S9 were colored with green or blue (D). (***E-F***) Estimated TF networks in macrophage lineages (A) and mast/killer lineages (B) s in *C. owczarzaki*, fly, sea urchin, tunicate, and mouse. (***G-J***) KEGG pathway analysis for genes estimated to have been expressed in ancestral blood cells, macrophages, and mast/killer cells. Fold enrichment scores for each infectious disease were shown in heat map (G). Triangle graphs show mean values ± SE of fold enrichment scores for bacterial, viral, and parasitic infection in ancestral blood cells (H), macrophages (I), and mast/killer cells (J). Mac, macrophage; Neut, neutrophil.

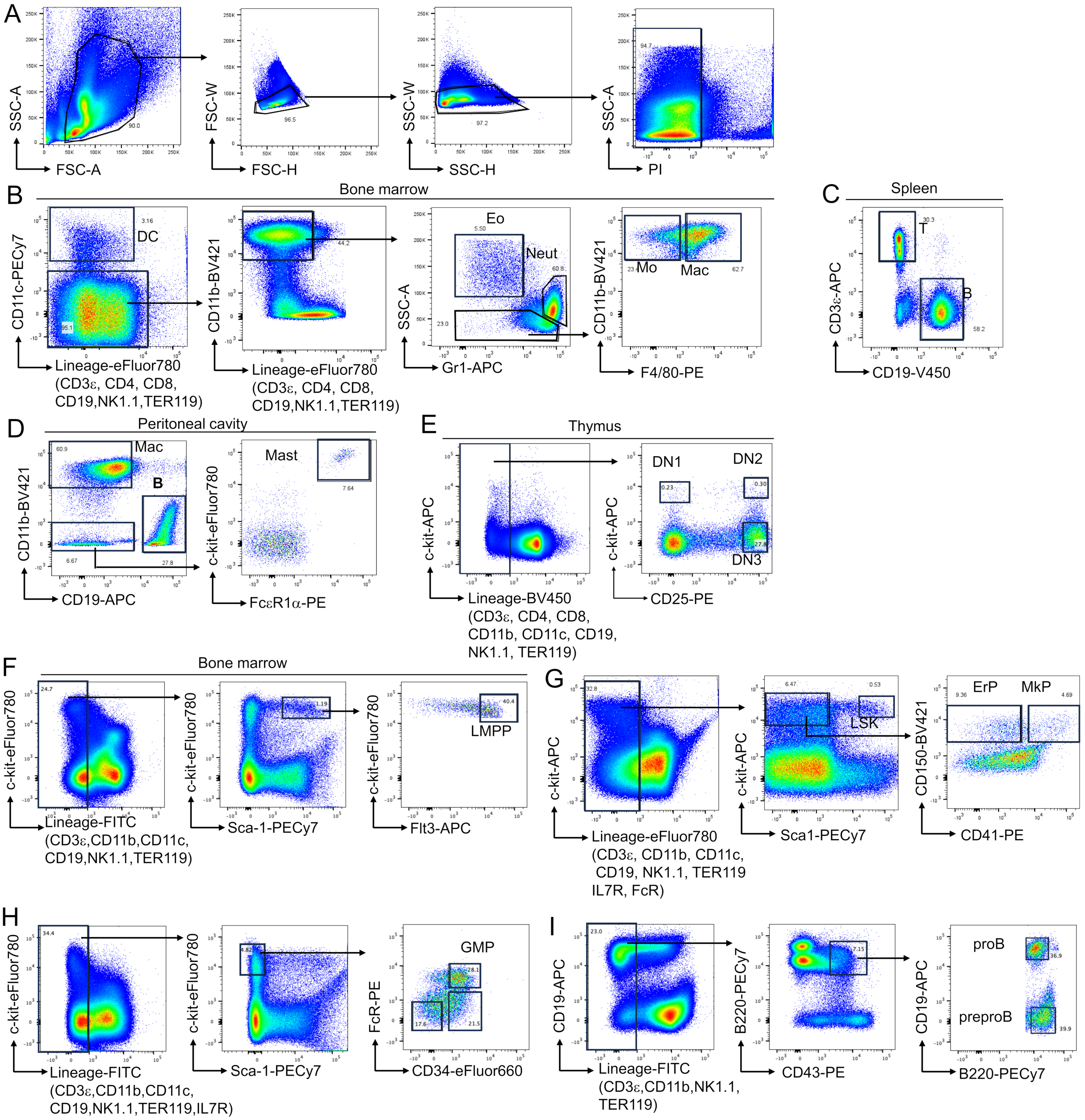

A
SSC-A
FSC-A
FSC-W
FSC-H
SSC-W
SSC-H
SSC-A
PI
B
Bone marrow
CD11c-PECy7
Lineage-eFluor780
(CD3ε, CD4, CD8,
CD19,NK1.1,TER119)
DC
CD11b-BV421
Lineage-eFluor780
(CD3ε, CD4, CD8,
CD19,NK1.1,TER119)
Eo
Neut
SSC-A
Gr1-APC
Mo
Mac
CD11b-BV421
F4/80-PE
C
Spleen
T
B
CD3ε-APC
CD19-V450
D
Peritoneal cavity
Mac
B
CD11b-BV421
CD19-APC
Mast
c-kit-eFluor780
FcεR1α-PE
E
Thymus
c-kit-APC
Lineage-BV450
(CD3ε, CD4, CD8,
CD11b, CD11c, CD19,
NK1.1, TER119)
DN1
DN2
DN3
c-kit-APC
CD25-PE
F
Bone marrow
c-kit-eFluor780
Lineage-FITC
(CD3ε,CD11b,CD11c,
CD19,NK1.1,TER119)
c-kit-eFluor780
Sca-1-PECy7
LMPP
c-kit-eFluor780
Flt3-APC
G
c-kit-APC
Lineage-eFluor780
(CD3ε, CD11b, CD11c,
CD19, NK1.1, TER119
IL7R, FcR)
LSK
c-kit-APC
Sca1-PECy7
ErP
MkP
CD150-BV421
CD41-PE
H
c-kit-eFluor780
Lineage-FITC
(CD3ε,CD11b,CD11c,
CD19,NK1.1,TER119,IL7R)
c-kit-eFluor780
Sca-1-PECy7
GMP
FcR-PE
CD34-eFluor660
I
CD19-APC
Lineage-FITC
(CD3ε,CD11b,NK1.1,
TER119)
B220-PECy7
CD43-PE
proB
preproB
CD19-APC
B220-PECy7

Fig. S8. Gating strategy for mouse blood cells. (***A***) Live singlets were gated, followed by the further strategies described below to identify various cell types. (***B-I***) Further gating strategy for dendritic cells, eosinophils, neutrophils, monocytes, and macrophages in bone marrow or spleen (B), for T and B cells in spleen (C), for macrophages, B cells, and mast cells in peritoneal cavity (D), for DN1-3 T-cell progenitors in thymus (E), for LMPPs in bone marrow (F), for MEPs, ErPs, and, MkPs in bone marrow (G), for GMPs in bone marrow (H), and proB cells and preproB cells in bone marrow (I). Eo, eosinophil; ErP, erythrocyte progenitor; GMP, granulocyte-monocyte progenitor; Mac, macrophage; MkP, megakaryocyte progenitor; Mo, monocyte; Neut, neutrophil.

A

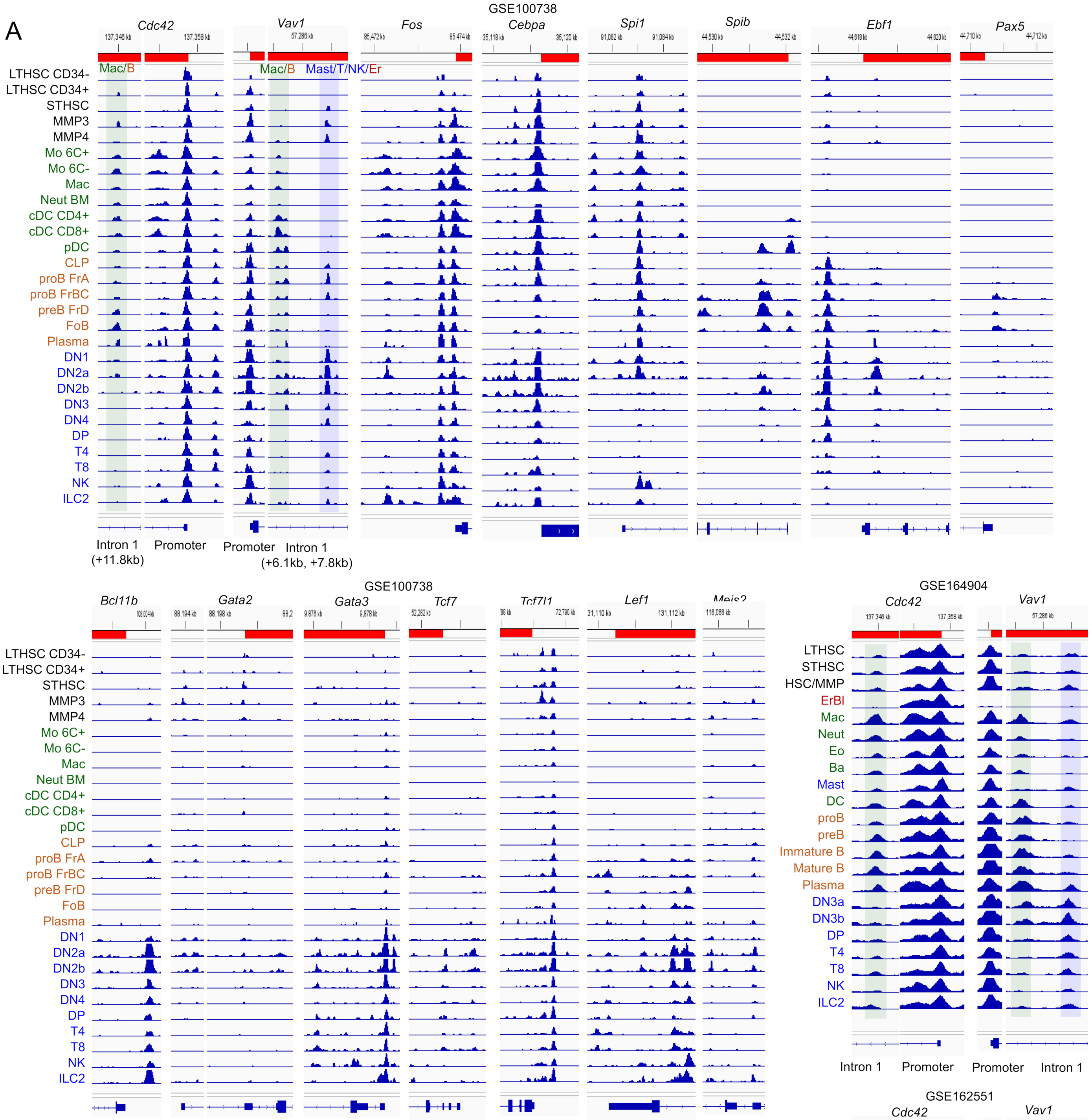

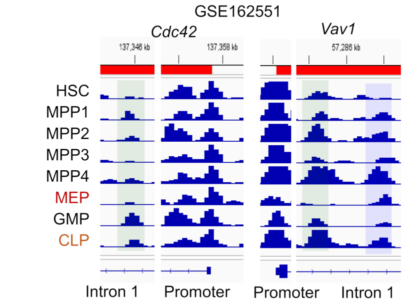

B

Log2(TPM+1)	Blood cell lineage data				Whole body cell lineage data
Gene Name	CV	Mean	1/CV rank	Mean rank	Blood vs non-Blood
Cox4i1	0.0482	10.65	7	28	-0.78
Cdc42	**0.0502**	**10.21**	**9**	**40**	**1.37**
Dazap2	0.0505	9.52	10	76	0.75
Srp14	0.0475	8.87	5	146	-0.09
Arf1	0.0448	8.72	1	179	0.12
Chmp2a	0.0455	8.50	3	214	-0.45
Rer1	0.0449	8.09	2	321	-0.23
Cope	0.0497	7.97	8	369	-0.47
Lman2	0.0461	7.38	4	702	0.29
BC031181	0.0481	7.27	6	795	-0.52
Vav1	**0.0897**	**7.79**	**668**	**464**	**6.20**

Fig. S9. Chromatin accessibility data for key TFs. (***A***) Chromatin accessibilities obtained from public ATAC-seq data (*SI Appendix*, Dataset S1) (56-58). ATAC peaks were normalized with peak highest at promoter region of *Cdc42* which was constitutively highly expressed across all the lineages. Promoter and neighbor regions of selected key TFs, *Cdc42*, and *Vav1* genes were shown. (***B***) List of 10 genes constitutively expressed with lowest coefficient of variation (CV) values among muse Mm2 blood data set which was related to ATAC-seq data. Among the 10 genes, *Cdc42* was second most highly expressed and also expressed higher than non-blood cells. *Vav1* gene is also listed.

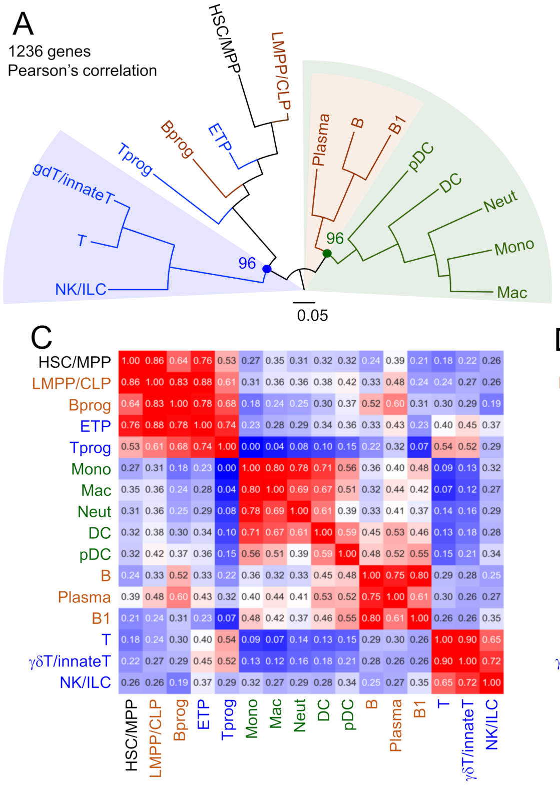

A
1236 genes
Pearson's correlation
HSC/MPP
LMPP/CLP
Bprog
ETP
Tprog
gdT/innateT
T
NK/ILC
Plasma
B
B1
pDC
DC
Neut
Mono
Mac
96
96
0.05
B
545 Orthogroups
Pearson's correlation
HSC/MPP
LMPP/CLP
Bprog
ETP
Tprog
gdT/innateT
T
NK/ILC
pDC
DC
Mac
Neut
Mono
Plasma
B1
B
94
94
0.05
C
D
HSC/MPP
LMPP/CLP
Bprog
ETP
Tprog
Mono
Mac
Neut
DC
pDC
B
Plasma
B1
T
γδT/innateT
NK/ILC
0.8
0.6
0.4
0.2
0.0

Fig. S10. Blood cell lineage classification with chromatin accessibilities. (***A-B***) Neighbour-joining phylogenetic trees of blood cell lineages based on chromatin accessibilities of 1236 genes (A) or 545 Orthogroups (B). Nodal support numbers of adjusted unbiassed bootstrap values at key bifurcations are shown with colored numbers and circles. The macrophage, mast/killer-cell, and B-cell clades are highlighted with green, blue, and orange background, respectively. (***C-D***) Pearson's correlation values between blood cell lineages based on chromatin accessibilities of 1236 genes (C) or 545 Orthogroups (D).

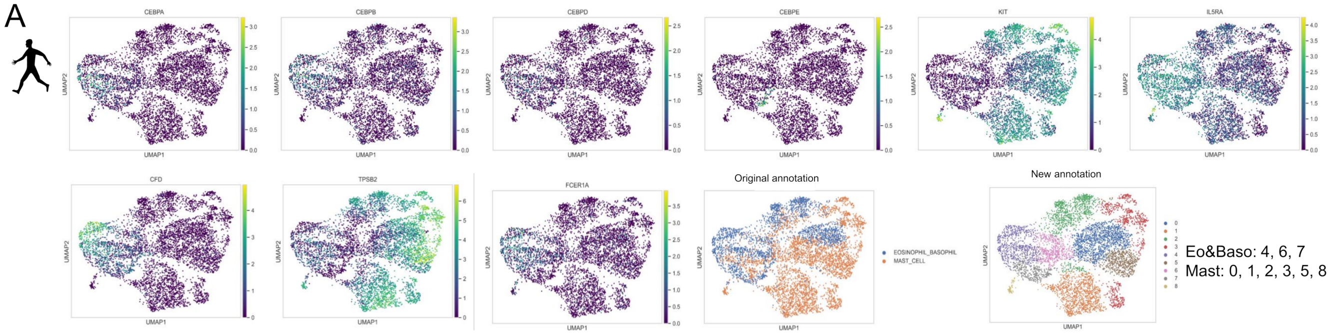

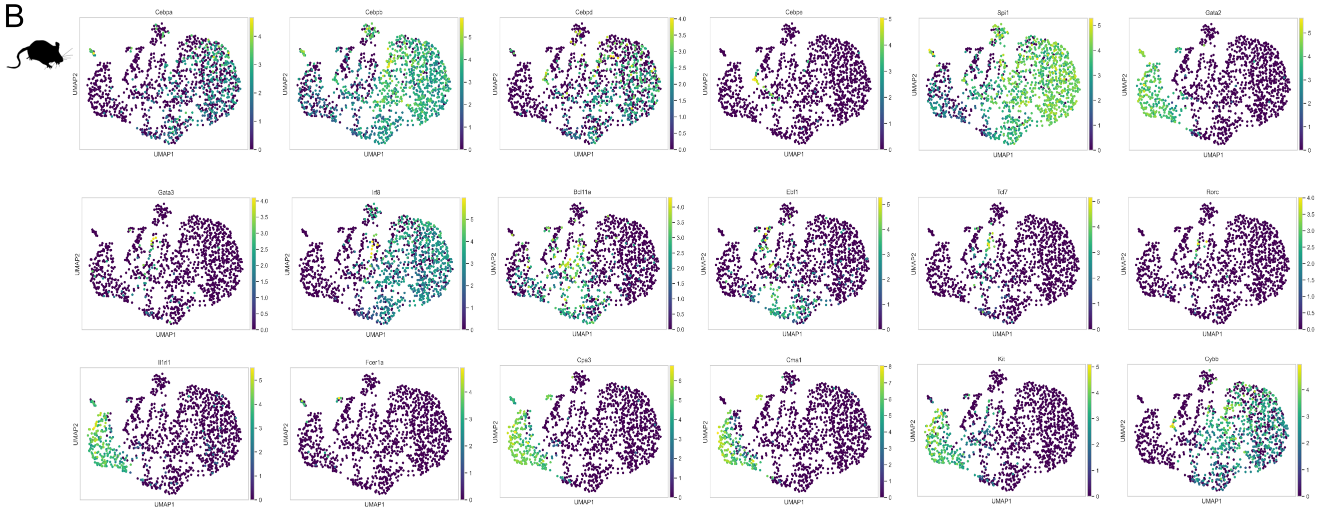

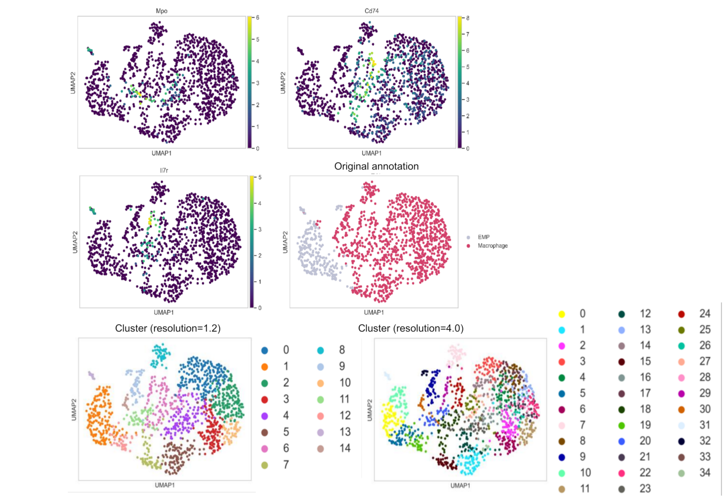

New lineage	Cluster (res=1.2)	Cluster (re=4.0)
EMP	11	any
Macrophage	0, 2-5. 8. 10	any
Macrophage	6	-24, -29
Macrophage	7	-15
Eosinophil	14	any
Basophil	13	any
MCP1	7	15
MCP2	1, 12	any
Mast	9	32
DC	6	24
B	9	9
ILC	6	29

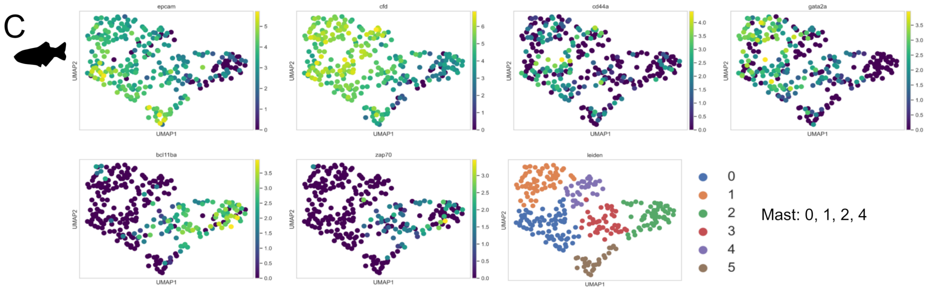

Fig. S11. Reanalysis of vertebrate single-cell RNA-seq data. (***A***-***C***) We reanalyzed human (A, Hs3), mouse (B, Mm4), and zebrafish (C, Dr2) single-cell RNA-seq data. For human data (A), we reanalyzed cells originally annotated as eosinophils/basophils and mast cells and annotated them based on representative genes. CEBPA/B/D/E^{+}, KIT^{-}, IL5RA^{+}, CFD^{+} cell clusters (#4, 6, 7) were regarded as eosinophil/basophil clusters, and CEBPA/B/D/E^{-}, KIT^{+}, TPSB2^{+} cell clusters (#0, 1, 2, 3, 5, 8) were regarded as mast cell progenitors. For mouse data (B), we reanalyzed cells originally annotated as macrophages and EMPs, and annotated lineages based on representative genes. Because these data contain small clusters of eosinophils, B cells, innate lymphoid cells, dendritic cells, and differentiated mast cells, we clustered them with two different resolution values. For zebrafish data (C), we reanalyzed thymic cells originally annotated as epithelial cells (original cluster 31) and annotated new clusters #0, 1, and 4 as mast cells based on representative genes.

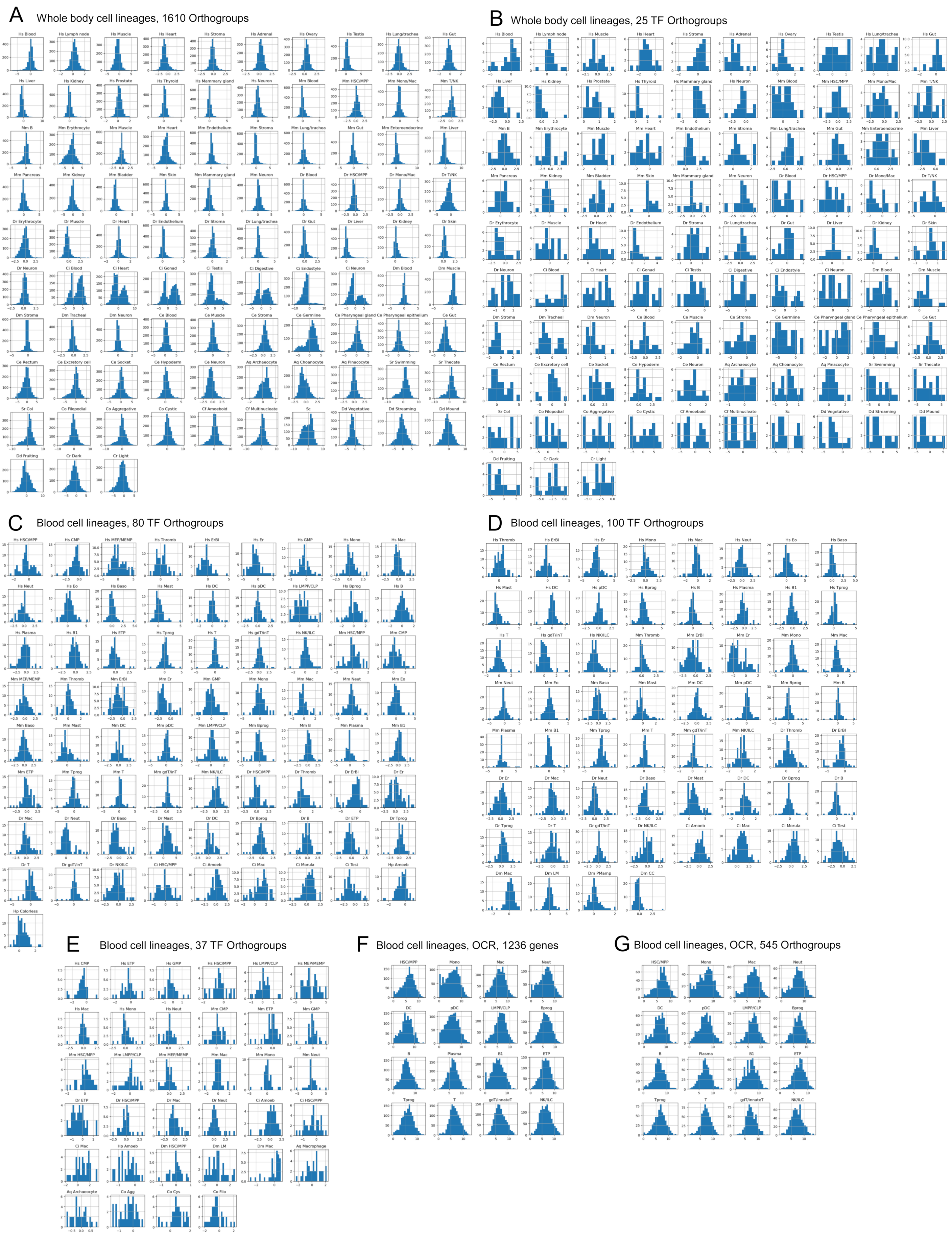
A Whole body cell lineages, 1610 Orthogroups
B Whole body cell lineages, 25 TF Orthogroups
C Blood cell lineages, 80 TF Orthogroups
D Blood cell lineages, 100 TF Orthogroups
E Blood cell lineages, 37 TF Orthogroups
F Blood cell lineages, OCR, 1236 genes
G Blood cell lineages, OCR, 545 Orthogroups

Fig. S12. Histograms of expression data. (***A*-*G***) Data distribution visualized with histograms for whole body cell lineages (A-B) and blood cell lineages (C-G). Each data is related to Fig. 1B (A), Fig. 1C (B), Fig. 2G (C), Fig. 2H (D), Fig. 2I (E), Fig. S10A (F), and Fig. S10B (G). Gene expression values (A-E) or open chromatin region (OCR) values (F-G) are shown. Aq, *Amphimedon queenslandica* (sponge); Ce, *Caenorhabditis elegans* (nematode); Cf, *Creolimax fragrantissima*; Ci, *Ciona robustra*, type A (*Ciona intestinalis*, tunicate); Co, *Capsaspora owczarzaki*; Cr, *Chlamydomonas reinhardtii*; Dd, *Dictyostelium discoideum*; Dm, *Drosophila melanogaster* (fly); Dr, *Danio rerio* (zebrafish); Hp, *Hemicentrotus pulcherrimus* (sea urchin); Hs, *Homo sapiens* (human); Mm, *Mus musculus* (mouse); Sc, *Saccharomyces cerevisiae* (yeast); Sr *Salpingoeca rosetta*.

Dataset S1 (separate file). List of sources of RNA sequencing data and amino acid sequences for homolog identification.

Dataset S2 (separate file). List of lineage annotations.

Dataset S3 (separate file). List of Orthogroups, TFs, phagocytosis related genes, and cell cycle related genes.

Dataset S4 (separate file). Representative codes for analysis.

Dataset S5 (separate file). List of Orthogroups for the analysis of whole body cell lineages

Dataset S6 (separate file). List of Orthogroups for the analysis of blood cell lineages

Dataset S7 (separate file). Data distribution normality

SI References

Sample References:

84. J. Choi et al., Haemopedia RNA-seq: a database of gene expression during haematopoiesis in mice and humans. Nucleic acids research 47, D780-D785 (2019).
85. Z. Li et al., Adult Connective Tissue-Resident Mast Cells Originate from Late Erythro-Myeloid Progenitors. Immunity 49, 640-653 e645 (2018).
86. P. He et al., The changing mouse embryo transcriptome at whole tissue and single-cell resolution. Nature 583, 760-767 (2020).
87. Q. Tang et al., Dissecting hematopoietic and renal cell heterogeneity in adult zebrafish at single-cell resolution using RNA sequencing. The Journal of experimental medicine 214, 2875-2887 (2017).
88. S. A. Rubin et al., Single-cell analyses reveal early thymic progenitors and pre-B cells in zebrafish. The Journal of experimental medicine 219 (2022).
89. S. Matsubara, T. Osugi, A. Shiraishi, A. Wada, H. Satake, Comparative analysis of transcriptomic profiles among ascidians, zebrafish, and mice: Insights from tissue-specific gene expression. PloS one 16, e0254308 (2021).
90. S. G. Tattikota et al., A single-cell survey of Drosophila blood. eLife 9 (2020).
91. S. Sogabe et al., Pluripotency and the origin of animal multicellularity. Nature 570, 519-522 (2019).
92. S. Kinjo, M. Kiyomoto, T. Yamamoto, K. Ikeo, S. Yaguchi, HpBase: A genome database of a sea urchin, Hemicentrotus pulcherrimus. Development, growth & differentiation 60, 174-182 (2018).
93. E. Ronnberg et al., Immunoprofiling Reveals Novel Mast Cell Receptors and the Continuous Nature of Human Lung Mast Cell Heterogeneity. Frontiers in immunology 12, 804812 (2021).
94. M. Mirdita, M. Steinegger, J. Soding, MMseqs2 desktop and local web server app for fast, interactive sequence searches. Bioinformatics 35, 2856-2858 (2019).
95. M. R. Hall et al., The crown-of-thorns starfish genome as a guide for biocontrol of this coral reef pest. Nature 544, 231-234 (2017).
96. A. Sebe-Pedros et al., Early metazoan cell type diversity and the evolution of multicellular gene regulation. Nature ecology & evolution 2, 1176-1188 (2018).
97. A. Sebe-Pedros et al., Cnidarian Cell Type Diversity and Regulation Revealed by Whole-Organism Single-Cell RNA-Seq. Cell 173, 1520-1534 e1520 (2018).
98. J. Sukumaran, M. T. Holder, DendroPy: a Python library for phylogenetic computing. Bioinformatics 26, 1569-1571 (2010).

99. H. Shimodaira, An approximately unbiased test of phylogenetic tree selection. Syst Biol 51, 492-508 (2002).
100. R. Suzuki, H. Shimodaira, Pvclust: an R package for assessing the uncertainty in hierarchical clustering. Bioinformatics 22, 1540-1542 (2006).
101. D. Castel et al., Dynamic binding of RBPJ is determined by Notch signaling status. Genes Dev 27, 1059-1071 (2013).
102. Y. Satou et al., A Manually Curated Gene Model Set for an Ascidian, Ciona robusta (Ciona intestinalis Type A). Zoological science 39, 253-260 (2022).
103. I. V. Kim et al., Chromatin loops are an ancestral hallmark of the animal regulatory genome. Nature 642, 1097-1105 (2025).
104. M. Ogasawara, T. Minokawa, Y. Sasakura, H. Nishida, K. W. Makabe, A Large-Scale Whole-Mount in situ Hybridization System: Rapid One-Tube Preparation of DIG-Labeled RNA Probes and High Throughput Hybridization using 96-Well Silent Screen Plates. Zoological science 18, 7 (2001).
105. J. E. Phillips, M. Santos, M. Konchwala, C. Xing, D. Pan, Genome editing in the unicellular holozoan Capsaspora owczarzaki suggests a premetazoan role for the Hippo pathway in multicellular morphogenesis. eLife 11 (2022).
